# Left-Wing Xenophobia in Europe

**DOI:** 10.3389/fsoc.2021.666717

**Published:** 2021-06-10

**Authors:** Svenja Kopyciok, Hilary Silver

**Affiliations:** ^1^ Department of Sociology, Brown University, Providence, RI, United States; ^2^ Department of Sociology, George Washington University, Washington, DC, United States

**Keywords:** xenophobia, left-right dimension, anti-immigrant attitudes, immigration, welfare chauvinism, nationalism, Europe, class politics

## Abstract

Given rising populist nationalism and multiplying meanings of “right” and “left,” this paper assesses whether Europeans who identify as extremely left-wing on the political spectrum hold anti-immigrant attitudes. In contrast to right-wing xenophobes, we further examine whether the political left, who conventionally emphasize class conflict, oppose immigrants less for cultural reasons and more for materialist reasons. We also consider whether socioeconomic status and values traditionally associated with the political left—favoring redistributive policies, egalitarianism, or social rights to benefits and services for immigrants—temper left- more than right-wing xenophobia. We find that a surprisingly large share of those who identify as far left do express extremely xenophobic attitudes, and we profile them in contrast to far right xenophobes. With logistic regression analysis of nine waves of the European Social Survey (2002–2018), we find that, all things equal, socioeconomic status influences far left xenophobia more than far right xenophobia, but inegalitarian values, less support for redistributive policies, and welfare chauvinism can only partially account for far left xenophobia and unexpectedly do not distinguish it from far right xenophobia. This implies that far left parties might adopt anti-immigrant policies to try to retain their loyal voters, even though such policies do not comport with broader left-wing values and may increase racial and ethnic inequality. Controlling for demographic and attitudinal differences reduces the probability of xenophobia among the far left by about sixty percent, but there remains some residual anti-immigrant attitudes among this group still to be explained.

## Introduction

“You have chosen to make the immigrant the scapegoat for the country’s problems rather than the financier who loots our country or the tax evader,” the far-left French leader Jean-Luc Mélenchon thundered from the National Assembly in July 2019. His La France Insoumise (Unsubmissive France) party offers an anti-capitalist critique of the European migration crisis, accusing the European Union’s border security policy of incentivizing both the exploitation of migrants and the fear of them. Such classically left-wing rhetoric combined Marxist economics, high-minded humanitarianism and populist blaming of the political class, the EU and global capitalism for inducing unwanted immigration in the first place (Migration Voter 2017).

But after the beheading of schoolteacher Samuel Paty in October 2020, Mélenchon blamed an entire group of migrants for the Chechen-origin assassin: “There is a very clear problem with the Chechen community in France. Chechens who are active in political Islam on social media must be found and expelled,” Mélenchon tweeted. “We are dealing with madmen and assassins who commit acts of Islamist terrorism which sully their religion and rot our lives.” (Alcoy 2020) His discourse this time drew upon cultural rhetoric, chauvinism, and Islamophobia.

In Greece, where the far-right Golden Dawn had staked out an anti-immigrant position, the far-left anti-austerity Syriza Party came to power in 2015 forming an improbable coalition with the Independent Greeks (ANEL), an anti-immigrant radical right-wing party. Yet, in opposition in 2020, Syriza leader Alexis Tsipras cast immigration as a nationalist and European issue to fend off Islam: “…we are up against a geopolitical threat. Not only because of the suffering of refugees, whom we must not treat as enemies and invaders, but from a geopolitical threat from Turkey. And when we have such a geopolitical threat, then all political forces ought to have, if not solidarity, then at least a framework for communication” (Vassilopoulos and Marsden 2020).

These examples illustrate ways that far left European politicians flirt with xenophobia.^1^ Anti-immigrant sentiments are widespread throughout Europe (Coenders et al., 2003). From the late 1980s until the turn of the 21st century, most studies show an upward trend in anti-immigrant attitudes in Europe as a whole, with considerable variation across regions and countries. East Europeans are more likely to oppose immigration than West Europeans despite smaller inflows (Ceobanu and Escandell 2010; Esipova et al., 2020). The Eurobarometer surveys show a steep rise in anti-foreigner sentiment from 1988 to 1994 and then a levelling off until 2000 (Semyonov et al., 2006) and even some decline from 2014 to 2016 (Dennison and Geddes 2019), but different cross-national studies report a mix of stable, decreasing, and increasing trends in anti-immigration attitudes (Ceobanu and Escandell 2010). From 2002 to 2016, countries varied in anti-immigrant trends, but in most countries, they were overall stable or slightly more positive, according to the European Social Survey and Pew Research (Heath and Richards 2016; Simmons et al., 2018; Dennison and Geddes 2019).

Since the 2015 refugee surge, however, there is some evidence of rising xenophobia. Between 2016 and 2019 in many European countries, there was a decline in Gallup’s Migration Acceptance Index based on whether people think migrants living in their country, becoming their neighbors, and marrying into their families are good things or bad things (Esipova et al., 2020). A Eurobarometer special survey of the EU28 (Eurobarometer 2018) found 17% of respondents had a “negative” perception of “the impact of immigrants on society.” Immigration became a more salient issue after the surge. Until summer 2020, when knocked to second place by the pandemic economic situation, Europeans considered immigration to be the most important issue facing the EU by far. The Autumn 2019 standard Eurobarometer report (European Commission 2019) shows that over one-third of the European public considered immigration to be the most important issue facing the EU, much surpassing the second most important, climate change (24%), but down from a peak of 58% in 2015.

Extreme right political parties are both enjoying and encouraging the spread of anti-immigrant opinion as they attract adherents with xenophobic tendencies. Is this a way to recruit people on the left? Are there Europeans who identify as left-wing who are also opposed to immigrants or immigration? If so, do they have different reasons for their opposition to newcomers than do those on the far right? This paper examines whether there is strong anti-immigrant sentiment among those who are on the far left and if so, how left-wing “xenophobes” may differ from those on the far right. Moreover, we examine whether these differences in characteristics influence left and right-wing xenophobia differently. We find that there is considerable xenophobia on the far left that is only partly accounted for by social insecurity, materialist, or cultural concerns.

Radical right parties are now defined largely by nativism, authoritarianism, and populism (Mudde 2007). The increasing electoral success of the far right over the past few decades is associated with the adoption of populist nationalist rhetoric and anti-immigrant policies, eclipsing in importance their early neo-liberal, anti-tax, anti-globalization stances and moving to the center on socioeconomic issues (de Lange 2007; Kitschelt 2007; Mudde 2007; Rydgren 2013; Oesch and Rennwald 2018). The previous radical right “winning formula” coalition (Kitschelt and McGann 1995) combining a neoliberal middle class with an anti-immigrant working class gave way to “welfare chauvinism,” promising the native working class generous social welfare benefits from which foreigners are excluded (Kitschelt and McGann 1995; Mudde 1999; Alonso and Fonseca 2012; de Koster et al., 2012).

Indeed, right-wing political parties use welfare chauvinism as an important element in their election campaigns (Ford and Goodwin 2014). Throughout Europe, the radical right have portrayed themselves as defenders of the welfare state and the native working class whom the left had abandoned (Fekete 2009). There is evidence too that mainstream parties adapt to populist parties on welfare chauvinism, but which parties adapt and when varies significantly (Schumacher and van Kersbergen 2016). Yet not all radical right-wing voters who believe immigrants place an unjustifiably high burden upon the welfare budget also hold affectively negative attitudes against them (Rydgren 2007). The belief that immigrants benefit more from the welfare system than they contribute to it only limits majority support for redistribution if political parties emphasize such claims, make them more salient, and politically activate them (Schmidt and Spies 2013).

All social classes are represented among far right voters, but working-class, blue collar, lower SES, and lower educated voters—groups that are thought of as the left’s natural constituency—are now consistently over-represented in the electorates of extreme right parties of Europe (Kitschelt and McGann 1995; Lubbers et al., 2002; Oesch 2008; Rydgren 2013; Harteveld 2016; Savelkoul and Scheepers 2017). Nevertheless, there is scant evidence to know whether these voters were previously nonvoters or whether they earlier supported traditionally left, right, or centrist parties (Browne et al., 2018). One might expect voters to defect from the moderate left–e.g., for adopting fiscally too conservative or socio-culturally too progressive positions like welcoming non-European immigrants. However, some very recent evidence shows that vote switchers from social democratic parties in Western and Northern Europe go predominantly to green and radical left parties, followed by mainstream right parties, not to far right-wing parties. Their rates of abstention and political demobilization are high, but an extremely small share of them ever swing to far right parties. Nor are the voters who leave social democratic parties primarily from the lower social classes. Social democratic parties have attracted voters whose parents were working class and who themselves are middle class, but whose partisanship is difficult to maintain (Häusermann et al., 2021).

Although the presence, strength, or nationalism of radical right parties do not appear to predict public opposition to immigration over time (Bohman and Hjerm 2016), and although right-wing voting is not only related to higher levels of anti-immigrant sentiment but to other matters as well (Wilkes et al., 2007), anti-immigration sentiment is possibly the most important individual attitude predicting a vote for a radical right party (Lubbers and Scheepers 2000; Lubbers et al., 2002; Norris 2005). Cross-national differences in support of extreme right-wing parties are particularly related to differences in public opinion on immigration (Semyonov et al., 2006). Over time, as pre-existing opposition to immigration is politically activated, the salience of immigration and voting for far right parties in most Western European countries become correlated (Dennison and Geddes 2019).

Indeed, the longstanding partisan cleavage over culture and religion increasingly cuts across the bipolar dimension of class-based conflict, buttressed by postmaterialist differences regarding environmentalism, feminism, religion, and gay rights (Kitschelt and McGann 1995; Inglehart and Norris 2016). While the traditional economic conflict of interests remains salient, every country seems to have some kind of cultural cleavage as well. These either reinforce or intersect with a distinct third dimension of political conflict over national identity, particularly as related to immigration, nationalism, and exclusionary, protectionist measures (Heath et al., 1999; Kriesi et al., 2006; Kitschelt and Rehm 2014; Heath et al., 2020). This makes it possible to speak of one right, two lefts, one being the traditional left that is still primarily defined by economic issues and the other, the new left, primarily defined by cultural stances. It is also possible to sketch a tripolar electoral system of a left, a moderate right and a radical right. Well-heeled professionals heavily support the left, large employers and managers the center-right, but the radical right entices small business and workers by opposing immigration, multiculturalism and European integration (Oesch and Rennwald 2018).

The response of left-wing parties to the anti-immigrant stance of extreme right parties, like welfare chauvinism, varies considerably across countries and time (Harmel and Svåsand 1997; Alonso and Fonseca 2012). That their constituency consists of progressive professionals *and* the working class poses a “progressive dilemma” (Goodhart 2004, 2013) for the political left, in which two progressive values – solidarity and diversity – collide. Whichever way they go, left parties may alienate some of their once loyal supporters. They can appeal either to privileged liberal egalitarian voters, who eschew ethnic nationalism and support cultural diversity, or to working-class voters, who benefit from redistributive policies and feel threatened by globalization and immigrant competition for jobs or benefits. In effect, the left must choose between the “winners” and “losers” from globalization and immigration (Kriesi et al., 2006; Harteveld 2016). Empirically, neither economic nor social globalization has a direct effect on welfare chauvinism, although there are some variations by social class (Mewes and Mau 2013). Supporting redistribution while restricting it to citizens are positions difficult for the left to reconcile with egalitarianism (Reeskens and van Oorschot 2012). Redistribution rests upon trust and shared nationhood, a contract between state and citizens that may be undermined by newcomers (Wimmer 1997). Nevertheless, it is hard to distinguish the economic interests behind welfare chauvinism – that is, unwillingness to share social benefits with foreigners – from simple prejudice (Leddy-Owen 2014).

Immigration and nationalism have scrambled the traditional class-based meanings of people’s ideological identifications as “left” and “right.” Although this conventional left-right “self-placement” scale has been the best single proxy for policy positions in European politics (Inglehart and Klingemann 1976; Huber 1989; Fuchs and Klingemann 1990), its meaning varies greatly by country, time period, and changing party systems. Left-right semantics have “an impressive absorptive power” (Knutsen 1995: 86). Since the 1980s, the dimension not only serves as shorthand for socioeconomic differences, but also for environmental and postmaterialist orientations and religious/secular values. Likewise, anti-immigrant attitudes and voting for right-wing parties in European politics are linearly related to self-placement on a right-to-left scale (Coenders and Scheepers 2003; Sides and Citrin 2007; Pichler 2010), and the effect of individuals’ left or right identity increased over time (Semyonov et al., 2006). This might imply that immigration has also been largely absorbed into the left-right dimension (van der Brug and van Spanje 2009), especially where the mainstream left Socialist and Social Democratic parties have supported immigration despite competition with extreme right parties.

Recent public opinion polls in eight European countries show that left-right ideological positions shape attitudes towards immigration more than do populist views (e.g., that elected officials do not care about ordinary people), but people with populist views are consistently more likely than their mainstream ideological counterparts to think immigrants have a negative impact on jobs and domestic security, with right-wing populists often being the most concerned about the effects of immigration (Simmons et al., 2018). In more comprehensive, universalist welfare states (Crepaz and Damron 2008) with less ethnic heterogeneity (Rapp 2016), there is less welfare chauvinism to restrict social benefits to citizens and less anti-immigrant resentment. This suggests that support for redistribution and egalitarianism as well as for immigrant social rights should temper anti-immigrant attitudes on the left.

As party competition has shifted from economic to cultural controversies, immigration has added non-economic significance to the right-left distinction. Working class voting for extreme right parties is not so much a consequence of changing political or economic preferences, since working class ideological orientation as left or right has not changed over time, as it is the reorientation of party competition providing an opportunity to activate those preferences. “The paradox of working-class support for the extreme right can be explained by the right-wing cultural views of these voters” (Spies 2013: 300). As (Lipset 1959 and 1981) documented decades ago, authoritarianism, populism, and xenophobia are widespread among the working class, regardless of economic preferences or identification as right or left. Left parties have largely ignored this uncomfortable reality, but workers might vote for the far right if that choice is available (Lubbers et al., 2002; Ivarsflaten 2005; Rydgren 2007; van der Brug and van Spanje 2009). Indeed, it is only where economic polarization is muted and a far right party activates xenophobia that working-class voters may act on their authoritarian, non-economic preferences and not on their left-wing economic interests.

So far, there is little research on the reaction of far left parties in particular to the far right challenge. In discussions of the intersection of cleavages, no parties seem to represent those who are left-wing on socio-economic issues and right-wing on cultural issues (van der Brug and van Spanje 2009). Yet left populism is becoming a more prominent feature of politics, especially in the poorer countries of South Europe where the economic downturn had a particular bite and where immigrants are nearby scapegoats (Bonikowski 2016). Some far left communist parties have indeed opposed immigration for what may be called materialist reasons, casting immigrants as exploited competitors for jobs or public benefits, defending workers’ *acquis sociaux*, and protecting citizens’ wages and taxes. Security and geopolitical justifications may also be invoked, as in the example at the outset of this paper. Given that nationalist populism is not the exclusive preserve of far right parties that go fishing for votes in the waters of their left-wing rivals, it is reasonable to hypothesize that there are potential voters that may be attracted by far-left anti-immigrant messages, too.

While anti-immigrant attitudes are indeed more widespread among those who identify as far right, we will show that they are not exclusively so. “There is no shortage of prejudice on the political left,” to put it succinctly (Sniderman et al., 2004: 69). Moreover, there are radical left populist parties in Europe as well as Latin America (March and Mudde 2005; March 2007; Mudde and Rovira Kaltwasser 2013; Kriesi 2014; Stavrakakis and Katsambekis 2014), although they have received less scholarly and media attention than right-wing populist parties. Far left parties have shifted from a class-oriented to a more populist rhetoric, railing against business, political, and professional elites who exploit “the people,” a collective that implicitly excludes, but does not mention immigrants and minorities. For right-wing populists, “the people” signifies the nation, but for left-wing populists, “the people” usually refers to a class of those socioeconomically downtrodden and victimized by self-interested elites like the global forces behind the Great Recession (Kriesi 2014). One study of elections across Europe between 1980 and 2016 found that support for right- and left-wing authoritarian populists had different motivations. Far right support did not respond to objective economic characteristics, while support for left-wing extreme populists – such as Syriza in Greece or Podemos in Spain, as well as the United Kingdom Labour Party under Corbyn – was sensitive to economic growth and unemployment rates (Rohac et al., 2017). For this reason, one would expect left-wing xenophobia likewise to reflect economic and material interests. Populism is less a thick, coherent ideology than a set of flexible ideas that can be used by left as well as right wing politicians (Laclau 1977; Bonikowski 2016), and so, may or may not include anti-immigrant or nationalist discourse.

Moreover, there are corresponding theories, many from the social psychology of prejudice, leading one to expect anti-immigrant attitudes on the left as well as the right (Quillian 2006). Regardless of party appeals, xenophobia may reflect individual (micro) variation more than contextual (macro) (Sides and Citrin 2007). These individual dispositions are usually classified as either economic and rational, or cultural and symbolic. The former refers to one’s objective or subjective economic status, material interests, or insecurities, and the latter to dispositions regarding one’s identity. Given conventional distinctions between left and right, one might hypothesize that xenophobes who consider themselves left-wing would respond more to socioeconomic insecurity, while those on the far right would feel their national or cultural identities are under threat from new immigrants.

Perceived competitive threats from newcomers or minorities, often measured by the size of the foreign-born population in a country or area, do appear related to anti-immigrant attitudes (Blumer 1958; Blalock 1970; Scheepers et al., 2002; Semyonov et al., 2006; Allport 2008). The (perceived) material competitive threat may come from the labor market – where immigrants supposedly take natives’ jobs or lower wages—or, in line with welfare chauvinism, from (perceived) undeservingness for, abuse of, or dependency on redistributive and social service programs for natives who are not working but who earlier paid taxes for these benefits. Accordingly, we expect individual anti-immigrant attitudes among members of the majority population to vary with perceived or actual social and economic vulnerability. Previous studies report that unemployed, lower income, and less educated individuals are more likely to express negative and hostile attitudes toward immigrants (Quillian 1995; Scheepers et al., 2002; Raijman et al., 2003; Hainmüller and Hiscox 2007; Pichler 2010).

Yet there is some evidence that individual opinions about immigrants are more concerned about the cultural threat than the perceived economic threat they pose (Sniderman et al., 2004; Sides and Citrin 2007; Schneider 2008). Therefore, in tandem with or independently of material interests, cultural predispositions – such as ethnic identities, religiosity, and postmaterialist values – may produce anti-immigrant sentiments due to perceived threats to national, religious, or racial community. Just as some find that a cultural backlash, combined with several social and demographic factors, provides the most consistent and parsimonious explanation for voting support for populist parties (Inglehart and Norris 2016), so too it may account for anti-immigrant sentiments. Nevertheless, it may be difficult to sustain the material/cultural distinction, since both types of interests and identities may simultaneously be at stake.

Group conflict or competitive threat theories are not the only explanations for xenophobia. Socially disengaged and distrustful individuals may fear foreigners more than socially integrated people do. Rather than juxtapose economic and cultural threat explanations for support of radical right and left parties, they can be combined as a problem of social integration or feelings of social marginalization more generally. Anti-immigrant attitudes are thus related to social exclusion, partially because of lower interpersonal trust (Pellegrini et al., 2021). Those who lack strong attachment to the normative order, a sense of social respect, or social engagement are more likely to be alienated from mainstream politics and to support radical parties (Gidron and Hall 2019).

Finally, there are demographics associated with anti-immigrant attitudes. Acceptance of migrants generally rises with education and income, and decreases with age, with those in the postmillennial generation the most accepting of all and traditionalists in the oldest generation the least accepting (Esipova et al., 2020). Resistance to integrating foreigners is stronger among manual workers, petit bourgeoisie, and the unemployed, at least in Germany (Coenders and Scheepers 2008). A similar demographic profile is found among supporters of populist parties across Europe, who tend to be older, male, less educated, and religious (Inglehart and Norris 2016).

Taken together, theories of group threat, social marginalization, and the authoritarian personality, as well as rising welfare chauvinism and working class voting for extreme right parties all lead to the expectation that there are extreme anti-immigrant sentiments on the far left. Moreover, populist parties have emerged at both ends of the political spectrum. We anticipate finding a curvilinear relationship between anti-immigrant attitudes and ideological orientation, with extreme xenophobes at each pole of the right-left scale. Further, we expect far left xenophobia exists independently of other demographic, socioeconomic, and attitudinal variables that are consistently related to anti-immigrant attitudes.

However, those on the far left who oppose immigrants may differ from those similarly xenophobic on the far right. We assess whether there are such differences in socioeconomic status, social vulnerability, or egalitarian attitudes, including towards redistribution and social rights, and whether they have different effects on left vs. right wing xenophobia. Materialist factors should be more important in predicting negative attitudes towards immigrants among the far left than the far right, given that leftist ideology emphasizes material-based class conflict. In contrast, given previous research presented above, we expect anti-immigrant sentiments on the right are more culturally or nationalistically motivated by identity threats than those holding such views on the left.

To what extent do those who identify with the political left oppose immigration? Do they resemble right-wing xenophobes? Can extreme left xenophobes reconcile their exclusionary attitudes towards immigrants with opinions associated with the left, such as support for social rights, government income redistribution or equality? This paper assesses these questions.

### Hypotheses

(1) Extreme xenophobia exists on both poles of the ideological spectrum.
(2) Left-wing xenophobia will differ significantly from right-wing xenophobia by emphasizing economic and material over cultural reasons for opposing immigrants. Left-wing anti-immigrant sentiments may reflect concerns about competition for jobs or welfare rather than identity or way of life.
(3) Traditionally left-wing values, such as egalitarianism, support for redistributive policies, or social rights to benefits and services for immigrants, will temper left-wing xenophobia more than they do right-wing xenophobia.

## Materials and Methods

### Data

We test these hypotheses with pooled data from the European Social Survey (ESS) waves 1–9 (2002–2018) (European Social Survey Cumulative File 2020). The ESS is a cross-national survey conducted biennially since 2002 to monitor changes in attitudes, beliefs, and behavior patterns of Europeans and is thus well suited for analyzing anti-immigrant attitudes on the continent.^2^ Thirty-six countries have participated in the survey since its first wave, 15 of which were included in all of the nine waves (Hungary, Slovenia, Poland, Great Britain, Ireland, Belgium, Netherlands, Germany, France, Spain, Portugal, Switzerland, Finland, Norway, and Sweden). The sample used in our analyses refers to all respondents 14 years and older from these 15 countries. The analyses are based on a total of 186,540 observations^3^ and are weighted with a combination of design weights and population size weights.^4^ Only the analyses of welfare chauvinism are based on a smaller sample (*N* = 41,618), as a question specifically measuring social rights to benefits and services for immigrants was only included in the rotating module of waves 4 and 8 (2004 and 2008).

### Methods

We are testing for a curvilinear relationship between political orientation and anti-immigrant attitudes, with higher xenophobia at both extremes of the political spectrum. We employ logistic regression to predict extreme xenophobia.^5^

In what follows, we first present descriptive statistics that contrast the extremely xenophobic far left and far right. We conduct difference-in-means tests to see if the two groups significantly differ on factors identified in the literature as demographic, socioeconomic, and cultural reasons for anti-immigrant sentiments. Adding to the basic logistic regression model, we then examine potential interaction effects. Based on those interaction effects, we calculate and present the predicted probability of being extremely anti-immigrant for the average person^6^ identifying as extremely left and extremely right. We examine whether those factors affect left and right-wing xenophobia significantly (*p* < 0.05) and if they have different effects for the two polar extremes. Lastly, we calculate how much all covariates of the model temper or moderate anti-immigrant attitudes among the far left and far right.

#### The Dependent Variable

Xenophobia is measured by whether the respondent thinks that immigrants make their country of residence a worse or a better place to live. The original 11-point scale variable was recoded into a dichotomous variable of being extremely anti-immigrant or not.^7^ Out of the entire sample, 3.62%, or 6,754 respondents, are extremely xenophobic. Overall, there was little change in these attitudes from 2002 to 2014, so pooling the waves should not pose a problem (Heath and Richards 2016).

#### Independent Variables: Measures of Political Ideological Orientation, Socioeconomic Status, Materialist Values, and Other Covariates

The respondent’s political ideological orientation is measured by his or her self-placement on the political left-right scale. We recoded the original 11-point scale into a 5-point right-left scale variable (extremely right, moderately right, centrist, moderately left, extremely left) in order to be able to show the hypothesized spike of extreme xenophobia among those who identify as extremely left, particularly as opposed to the moderate left.^8^ In total, 2.85% of the sample identify as extremely right and 3.11% as extremely left. The three largest ideological groups are the centrists (23.52%), the moderate right (31.92%), and the moderate left (29.6%).

Several variables are added to the model in order to assess whether socioeconomic status as well as values typically associated with the political left temper left-wing xenophobia and have a stronger impact on left-wing than right-wing xenophobia. First, we control for objective and subjective socioeconomic status by including information on the respondent’s education (post-secondary degree), labor force status (out of the labor force, unemployed, employed), and whether s/he feels socioeconomically vulnerable (finding it difficult to live on the current household income).^9^ In order to control for values typically associated with the political left, we include measures of support for income redistribution (dummy), the importance the respondent assigns to equal treatment and opportunities (very important, important, somewhat important, not important), and of welfare chauvinism (dummy). We define welfare chauvinism as responding “never” to the question “When should immigrants obtain the same rights to social benefits and services as citizens already living here?”

In addition to these main variables of interest, we control for a variety of personal characteristics found to be associated with anti-immigrant attitudes. The model controls for several variables that might induce people to perceive immigrants as a cultural threat, such as the importance respondents ascribe to following traditions and customs (very important, important, somewhat important, not important) and self-reported religiosity (11-point scale from 0–10). To measure respondents’ social marginality, insecurity, or lack of social capital, we include covariates for social trust (trust in other people: distrustful, neutral, trusting), social integration (less, same, or more social activities than others of the same age), and political participation (a dummy of having voted in the last national elections). Populist tendencies were controlled for with distrust in politicians (no trust at all, distrustful, neutral, trusting). Finally, the model includes sociodemographic information (age, gender, and migration background), and year and country in order to control for unobserved effects of time and geographic differences.^10^

## Results

### The Distribution of Anti-Immigrant Attitudes by Political Ideology

**FIGURE 1:**
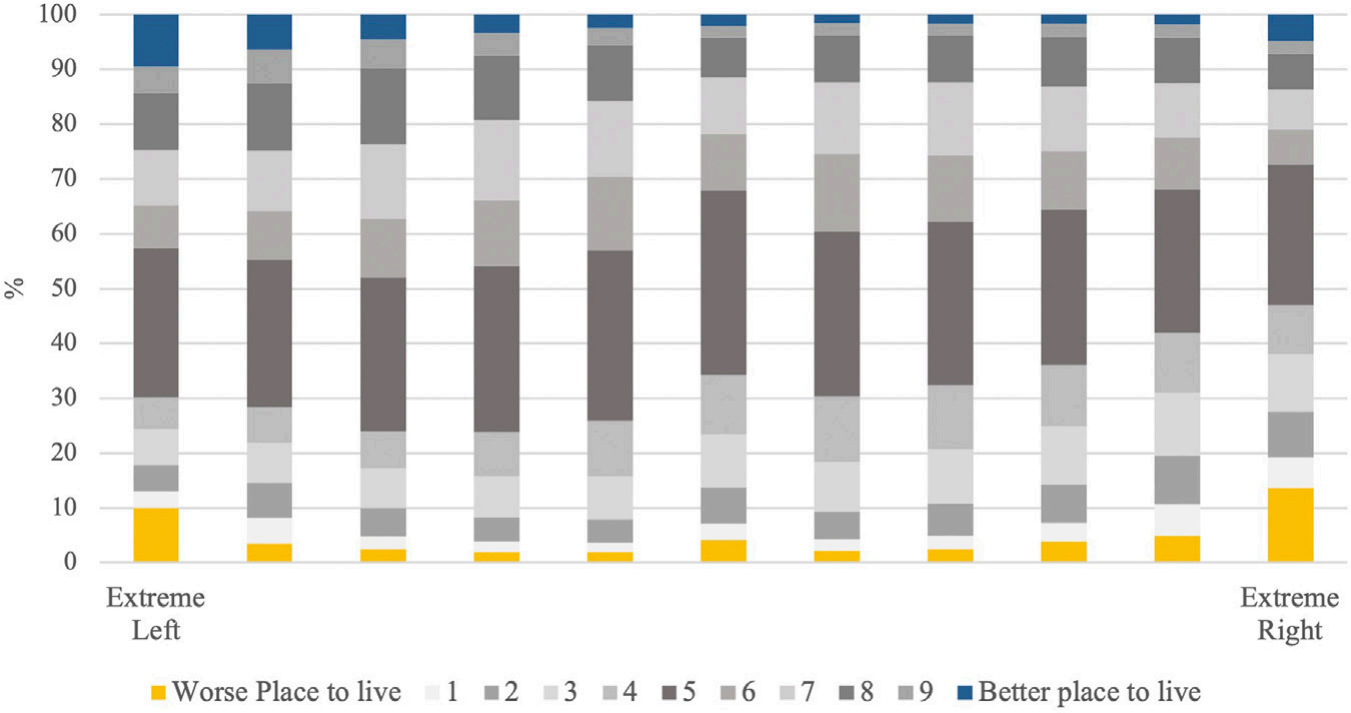
Relationship between Political Ideology and Attitudes Towards Immigrants. Source: European Social Survey, cumulative dataset, waves 1–9. Own calculations.

**FIGURE 2:**
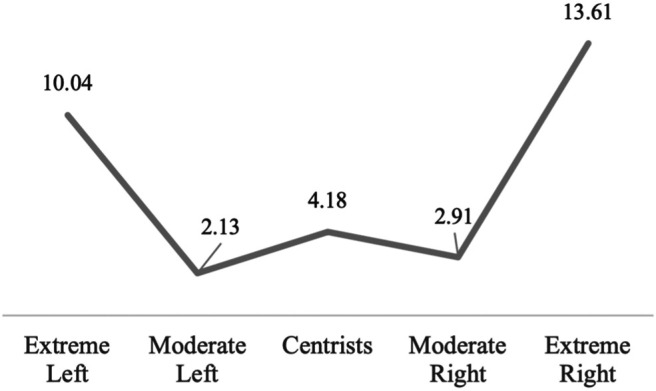
Percentage of Extremely Anti-Immigrant Respondents by Political Ideology. Source: European Social Survey, cumulative dataset, waves 1–9. Own calculations.

Figure 1 shows how attitudes towards immigrants differ by political ideology. As expected, the majority of respondents have a relatively moderate opinion towards immigrants. There is, however, a substantial subgroup within the far right and the far left with extremely negative attitudes towards immigrants. The relationship between political orientation and xenophobia is thus not linear as often assumed, but curvilinear with the extremes being more xenophobic than those in between (see also Figure 2). Ten percent of the far left are extreme xenophobes, more than any other ideological grouping except for the far right. It is even slightly higher than the 9.5% of the far left with extremely pro-immigrant attitudes.

The prevalence of extreme xenophobia among the extreme left is closer to that of the extreme right (3.5% difference) than to the prevalence among the other more centrist political groupings. It is particularly different from that of the moderate left; the difference is almost 8 percent. That said, besides this higher incidence of extremely negative attitudes towards immigrants on the extreme left, there also is a large number of far left respondents with extremely positive attitudes towards immigrants (9.5%). In other words, those who identify as extremely left are highly polarized regarding immigration issues. However, those who claim to be on the extreme right are similarly, if slightly less, polarized. There is a larger share of those on the far right with extremely pro-immigrant attitudes than there is among the moderate right, centrists, and the moderate left. This implies that opinions about immigration are not necessarily “absorbed” by the right-left dimension, so that even at the extremes of the ideological spectrum, there are divergent attitudes towards immigrants.

**FIGURE 3:**
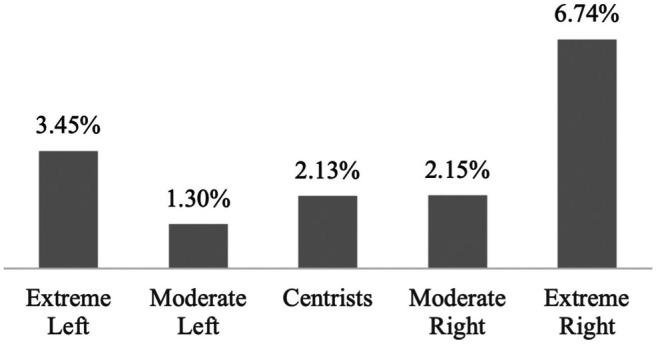
Predicted Probabilities of Being Extremely Xenophobic by Political Ideology. Source: European Social Survey, cumulative dataset, waves 1–9. Own calculations.

Results from the logistic regression analysis reveal that, even when controlling for various factors known to influence anti-immigrant attitudes, the uptick of strong anti-immigrant attitudes on the extreme left of the political spectrum persists (see Figure 3). The predicted probability of being extremely xenophobic for the average person of the sample identifying as extremely left is 3.45%, compared to between 1.3 and 2.15% for those identifying as moderately left, moderately right, or centrist. As expected, it is highest when identifying as extremely right (6.74%).^11^

**FIGURE 4:**
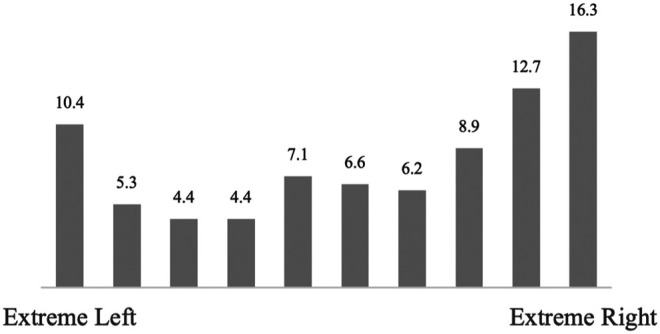
Percentage of Respondents Strongly Agreeing that “Immigrants Are Very Bad for the Development of the Country” by Political Ideology. Source: European Values Study 2017, Integrated Dataset, wave 7. Own calculations.

Data from the 2017 wave of the European Values Study (European Values Study 2020) confirms this high incidence of anti-immigrant sentiment on the extreme left of the political spectrum: 10.4% of those identifying as extremely left think that immigrants are very bad for the development of their country, while only 4.4–5.3% think so among the moderate left (see Figure 4). The pattern is even stronger regarding the perceived effect of immigrants on the welfare system: 26.5% of the extreme left thinks that immigrants are a strain on the welfare system, compared to 6–7% of the moderate left.^12^ The curvilinear relationship is similarly evident for immigrants taking jobs. Unexpectedly, both the far left and the far right are more likely to agree that immigrants should not maintain their distinct customs and traditions. Thus, both material and cultural justifications for anti-immigrant attitudes are found at the extreme left of the political spectrum.

To summarize, there are indeed individuals who identify themselves as being on the extreme left of the political spectrum who are nevertheless extremely anti-immigrant. Who constitutes this small, very xenophobic group among the extreme left? How do their characteristics differ from those of the extremely xenophobic far right, and which factors can account for their negative attitudes towards immigrants?

### Characteristics of the Extremely Xenophobic Extreme Right and Extreme Left

Before we compare the extremely xenophobic far left and far right to each other, we contrast them to the average characteristics of the overall sample in order to illustrate some essential differences between extreme xenophobes and the average person in the sample (see Table 1).

**TABLE 1:** Average group characteristics of the extremely xenophobic far right, far left and the overall sample.

	Xenophobic far right			Xenophobic far left		Overall sample	
	*Mean*	SD		*Mean*	*SD*	Mean	SD
*Sociodemographic Background*							
Age	53.53	18.61		54.67	16.83	50.05	17.33
Male	53.39			49.06		48.88	
1st/2nd generation immigrant	11.20			10.29		12.85	
*Socioeconomic Status*							
Post-secondary degree	12.31		^a^	9.43		31.43	
Labor force status			^c^				
Out of the labor force	53.11			58.66		42.42	
Employed	42.19			34.31		53.99	
Unemployed	4.70			7.03		3.60	
Finding it difficult on current household income	30.98		^d^	40.99%		17.46	
*Attitudes Towards Income Redistribution*							
Support of income redistribution	74.97		^d^	84.56		72.25	
Egalitarianism			^d^				
Very important	33.47			46.83		32.85	
Important	32.23			31.39		43.27	
Somewhat important	23.51			15.44		21.08	
Not important	10.79			6.35		2.80	
Welfare chauvinist	39.74		^b^	27.78		5.25	
*Cultural Values*							
Importance of following traditions and customs			^d^				
Very important	38.31			30.87		16.89	
Important	30.43			26.76		32.39	
Somewhat important	20.89			24.01		36.90	
Not important	10.37			18.35		13.83	
Subjective religiosity (0-not religious to 10-very religious)	4.99	3.63	^d^	3.78	3.42	4.54	2.97
*Social Capital, Trust, and Integration*							
Trust in people			^c^				
Distrustful	58.09			67.92		31.61	
Neutral	19.36			16.64		19.82	
Trusting	22.54			15.44		48.56	
Level of social activity			^c^				
Less than others	47.03			55.23		36.58	
About the same	35.68			33.28		45.38	
More than others	17.29			11.49		18.05	
Voted in last national elections	81.60		^d^	72.04		81.85	
Trust in politicians			^c^				
No trust at all	47.58			57.63		11.78	
Distrustful	31.54			26.24		45.08	
Neutral	9.41			8.92		18.42	
Trust politicians	11.48			7.20		24.72	
N (total, waves 1–9)	723			583		186,540	
N (total, waves 4 and 8, analysis of welfare chauvinism)	156			126		41,618	

Source: European Social Survey, cumulative dataset, waves 1–9.

Notes: Two-tailed t-tests and chi-square tests were conducted as comparison in means tests.

*P*-values:

a *p* < .1,

b *p* < .05,

c *p* < .01.

d *p* < .001.

#### Xenophobic Extreme Left and Extreme Right Versus the Overall Sample

As others have found, the xenophobic extreme left and right in our sample are older than the average European in the sample. Both groups are socioeconomically more disadvantaged, objectively and subjectively. They have lower levels of education, are more likely to be out of the labor force and to be unemployed. Accordingly, they feel much more socioeconomically insecure, reporting greater difficulty living on their household income. In line with economic, not cultural understandings of left and right, left-wing xenophobes have more egalitarian views than the overall sample and are more supportive of income redistribution. Surprisingly egalitarian views are also relatively prevalent among extreme right-wing xenophobes, only slightly less so than among the overall sample. Despite this high level of egalitarianism at both ideological extremes, the far left and far right are significantly more likely than the overall sample to be welfare chauvinists. While only 5% of the entire sample would never grant immigrants social benefits and services, 28% of left-wing xenophobes and 40% of those on the right would never do so. This suggests that parties aiming to win votes among xenophobes on the far left might appeal to their welfare chauvinism.

Those at both ideological poles are culturally more conservative, attributing importance to traditions and customs. Which traditions and customs they have in mind is unclear. As expected, left-wing xenophobes are less, and right-wing xenophobes more religious than the overall sample. In addition, they have less social capital: they have lower levels of social trust and are less socially and politically active. They have populist tendencies, being less likely to trust politicians. Among the xenophobic extreme left, this lack of trust is reflected in a lower voter turnout compared to the overall sample. The xenophobic extreme right, in contrast, is not less likely to vote than the overall sample.

#### Extremely Xenophobic far Left Versus Extremely Xenophobic far Right

**FIGURE 5:**
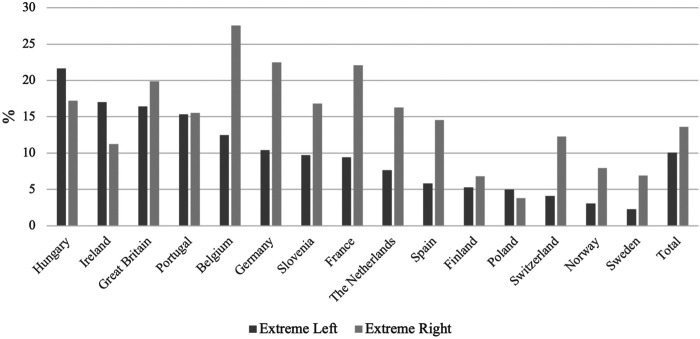
Extreme Xenophobia Among the Far Left and Far Right by Country. Source: European Social Survey, cumulative dataset, waves 1–9. Own calculations.

We examined the pattern of extreme left and right-wing xenophobia by country. There are no obvious national characteristics, such as economic conditions, size of the immigrant population, region, welfare regime, or presence of a populist party to explain the cross-country variation (see Figure 5). Hungary, Ireland, and Poland are outliers with self-professed left-wing xenophobia being more prevalent than right-wing xenophobia.

The extremely xenophobic far left and far right are similar in many characteristics, but they also significantly differ in others. First of all, there is no significant difference in their average age, as well as their sex and native-immigrant ratio. Despite these sociodemographic similarities, extremely xenophobic left-wing respondents are significantly more objectively and subjectively socioeconomically disadvantaged than xenophobes on the extreme right of the political spectrum. They are more likely to be out of the labor force and unemployed and 40% say that they find it difficult to live on their present income, compared to 30% of extreme right-wing xenophobes. The two groups’ average levels of education, in contrast, only marginally differ, with the xenophobic extreme left having a slightly lower average educational level.

Support for the redistributive welfare state is traditionally a defining policy position of the political left. Accordingly, almost 85% of individuals who identify as far left and who are xenophobic still support income redistribution. Among the xenophobic extreme right, support is lower at 75%, but also surprisingly high. In addition to support for redistributive measures, the xenophobic far left also consider equal treatment and opportunities to be very important, even though their anti-immigrant propensity may suggest otherwise. Forty-seven percent of extreme left xenophobes are strongly supportive of egalitarian ideals, which is not only significantly more than among extreme right xenophobes (33.5%), but also more than among the entire sample (33%).

How can xenophobes reconcile their apparent solidarity with their negative attitudes towards immigrants? Support of income redistribution does not necessarily reflect egalitarian views. It may result from self-interest; one may support redistributive measures for his/her own benefit as well as to offer other people more opportunities. Insofar as fewer xenophobic respondents on the extreme left are employed, they may be more dependent on welfare benefits, which may make them more self-interested supporters of those measures. At the same time, they are also stark defenders of egalitarian values. Perhaps they imagine hoarding those equal opportunities for themselves.

To get a better sense of whether left and right-wing xenophobes support redistributive measures primarily out of self-interest, and how their attitudes towards those measures are related to their attitudes towards immigrants, we compare their levels of welfare chauvinism–whether they want to draw ethnic boundaries around the welfare state and restrict social benefits to natives. As mentioned, both left and right xenophobes are indeed much more willing to exclude immigrants from state redistributive measures than the average person in the sample. Welfare chauvinism is relatively high among far left-wing xenophobes (28%) and even significantly higher among the extreme right (40%). This suggests that welfare chauvinism and anti-immigrant attitudes are closely related, and that some respondents at both political poles support income redistribution out of self-interest, but significantly more so among the extreme right than among the extreme left. Egalitarian values traditionally associated with the left hardly tempers anti-immigrant attitudes among the far left, as the percentages of supporters of redistributive measures and of respondents with egalitarian values are relatively high.

As expected, xenophobes on the extreme right are more culturally conservative than those on the extreme left, which may make them more likely to perceive immigrants as a cultural threat. Compared to the extreme left xenophobes, they find it significantly more important to follow traditions and customs and they are significantly more religious. Both xenophobic groupings, however, value customs and traditions much more than the average respondent. Symbolic boundaries, such as cultural values and traditions, do seem related to anti-immigrant attitudes both on the extreme right and the extreme left of the political spectrum.

In line with the xenophobic extreme left’s self-reported socioeconomic insecurity, they also report less social integration than extreme right-wing xenophobes. They have very low levels of social trust. The general distrust of other people is also reflected in their populist distrust of politicians, which is extremely high compared to the overall sample and surprisingly, significantly higher than that of the xenophobic extreme right. Accordingly, fewer extreme left xenophobes are politically engaged; significantly fewer left-than right-wing xenophobes voted in the last national elections.

### The Role of Material Factors in Left and Right-Wing Xenophobia

We hypothesized that material interests would play an important role in left-wing xenophobia, whereas cultural and national identities and values may be more decisive for attitudes towards immigrants among the extreme right. What is defined as “left” is traditionally based on economic factors and interests, such as social class and support of the welfare state. As many individuals who identify as left are workers, they may be more inclined to perceive immigrants as an economic threat to their jobs and income stability, as many unskilled immigrants compete for the same jobs. Moreover, the extremely xenophobic far left is more socioeconomically disadvantaged than the xenophobic extreme right, so it may also be more likely to perceive immigrants as an economic threat. At the same time, values traditionally associated with the left, such as income redistribution and egalitarianism, may temper those fears and negative attitudes towards immigrants. Due to the political left’s traditional egalitarianism and solidarity, left-wing xenophobia may be less influenced by cultural factors, such as perceiving immigrants as a threat to one’s cultural or national identity.

#### Perceived Cultural and Economic Consequences of Immigration Among the far Left and far Right

One way to test these hypotheses is to look at whether the extreme left and extreme right differ in their opinions on the economic and cultural consequences of immigration. There are two questions in the European Social Survey core module that measure the perception of immigrants as an economic and cultural threat. One assesses whether the respondent thinks that immigration is bad or good for the country’s economy, and the other, whether the country's cultural life is undermined or enriched by immigrants. Both are measured by an 11-point scale of 0–10 (0 = undermined/bad for the economy and 10 = enriched/good for the economy). A comparison of means of those two forms of xenophobia reveals that the extreme left is indeed significantly more xenophobic economically than culturally than the average European. But so is the extreme right! Both political extremes have a significantly higher mean of agreement with the statement that immigration is bad for the country’s economy than they do with the statement that cultural life is undermined by immigrants (*p* < 0.001). However, the difference in means between economic vs. culture-based xenophobia is larger among the extreme left than among the extreme right. We thus find partial support of our hypothesis. Material considerations play a decisive role for anti-immigrant attitudes of both the far left and the far right, while cultural factors are less decisive in the xenophobia of the extreme left than of the far right.

#### The Effect of Socioeconomic Status on Extreme Xenophobia

Next, we examine the moderating effect of socioeconomic status on extreme left and right-wing xenophobia. After running the basic logistic regression model predicting extreme xenophobia (see Table 2), we sequentially test for potential interaction effects and present the predicted probabilities of being extremely xenophobic for the extreme left and right, respectively, with additional characteristics.^13^ We begin with measures of socioeconomic status: post-secondary education, labor force status, and perceived socioeconomic position. A comparison of the predicted probabilities of being extremely xenophobic when the average person identifies as extremely left and does or does not have a post-secondary degree reveals that lower levels of education increase the chance of being extremely xenophobic from 1.5 to 3.6%. Among the far right, the predicted probability of being extremely xenophobic is not statistically different by level of education.

**TABLE 2:** Results from logistic regression on extremely anti-immigrant attitudes (Odds ratio).

	*Odds Ratio*	*SE*
Political ideology (ref.: Centrists)		
Extreme right	3.33^d^	0.25
Moderate right	1.01	0.05
Moderate left	0.61^d^	0.03
Extreme left	1.65^d^	0.12
Age	1.01^d^	0.00
Male	1.03	0.04
1st/2nd generation immigrant	0.66^d^	0.04
Post-secondary degree	0.47^d^	0.03
Labor force status (ref.: Out of the labor force)		
Employed	0.89^b^	0.04
Unemployed	1.29^c^	0.11
Finding it difficult on current household income	1.64^d^	0.07
Trust in people (ref.: Distrustful)		
Neutral	0.67^d^	0.03
Trusting	0.51^d^	0.02
Level of social activity (ref.: Less than others)		
About the same	0.85^d^	0.04
More than others	0.83^d^	0.05
Religiosity, 0 (not religious) to 10 (very religious)	0.96^d^	0.01
Importance of following traditions and customs (ref.: Very)		
Important	0.65^d^	0.03
Somewhat important	0.48^d^	0.03
Not important	0.60^d^	0.04
Importance of egalitarianism (ref.: Very)		
Important	0.99	0.05
Somewhat important	1.25^d^	0.07
Not important	2.44^d^	0.21
Support income redistribution	1.06	0.05
Trust in politicians (ref.: No trust at all)		
Distrustful	0.24^d^	0.01
Neutral	0.19^d^	0.01
Trusting	0.12^d^	0.01
Political participation (voted in last national election)	0.80^d^	0.04
*Pseudo R* ^*2*^	0.1913	
*N*	186,540	

*Source*: European Social Survey, cumulative dataset, waves 1–9.

Notes: The model also controls for year and country. SE = Standard Error.

*P*-values:

a *p* < .1,

b *p* < .05,

c *p* < .01,

d *p* < .001.

Other things equal, labor force status marginally affects an extremely left-wing person’s chance of being xenophobic (*p* < 0.1). If the average person in the sample identifies as extremely left, s/he has a higher chance of being extremely xenophobic when s/he is out of the labor force (4.3%) than if s/he is employed (2.9%). In contrast, labor force status does not significantly affect extremely negative attitudes towards immigrants of a far right person. Far left and far right xenophobes are both more likely to feel socioeconomically vulnerable than the average respondent, extreme left xenophobes even more so than those on the extreme right of the political spectrum. Accordingly, perceived economic difficulty affects extremely anti-immigrant attitudes among both ideological groups, even when all other covariates are controlled. The effect among the extreme right is, however, only marginally significant (*p* < 0.1). Those who find it difficult to live on their current household income have a statistically significantly higher chance of being extremely xenophobic than those who do not report that difficulty. The predicted probability increases from 3.3 to 4.9% among the extreme left and from 6.5 to 9.1% among the extreme right.

Like socioeconomic insecurity, feeling socially insecure has a stronger effect on extreme xenophobia among the far left than the far right. When the average person identifies as left-wing and feels less socially active than other people his/her age, s/he is marginally more likely to be xenophobic than if s/he sees her- or himself as more socially active than others. Self-perceived social activity, in contrast, does not have a statistically significant effect on extreme right-wing xenophobia. Similarly, low levels of social trust increase the probability of being extremely xenophobic among the far left but is only marginally significant among the far right. Vertical trust, or trust in politicians, has a significant effect on extremely anti-immigrant attitudes among both the extreme left and right. Low levels of trust strongly increase the chance of being extremely xenophobic for those at both ends of the ideological divide, even more so among the far right than the far left. This distrust of political elites, which is a hallmark of populism, is manifested in xenophobia at both ends of the political spectrum.

To summarize, we hypothesized that socioeconomic status is more decisive for left- than for right-wing xenophobia. We found all three socioeconomic status indicators and social insecurity had an effect on left-wing xenophobia, although labor force status had only a marginal impact. In contrast, among the extreme right, labor force status did not affect one’s chance of being extremely xenophobic, and subjective feelings of one’s socioeconomic position only marginally did so.

### The Effect of Values Traditionally Associated with the Left on Left and Right-Wing Xenophobia

The previous analyses offer some support that, other things equal, socioeconomic status plays a more important role in left-than right-wing xenophobia. How much do socioeconomic values traditionally associated with the left temper anti-immigrant attitudes among the extreme left? On the one hand, being out of the labor force increases the chance of being extremely xenophobic of those on the far left, which suggests that they may perceive immigrants as a threat to the welfare system and the social benefits they may depend on. On the other hand, typically left values, such as egalitarianism and support for the welfare state, may temper these materially self-interested reactions to immigrants.

#### The Effect of Support of Income Redistribution

The comparison of means revealed that the extremely xenophobic far left strongly supports income redistribution and, as expected, more so than the xenophobic far right. However, left-wing xenophobes are significantly less likely to support redistribution than those who identify as far left and are not extremely xenophobic (88 vs. 85%). Accordingly, other things equal, the predicted probability of being extremely xenophobic decreases from 5.2 to 3.2% when the average person identifies as extremely left and supports income redistribution, but the effect is only marginally significant (*p* < 0.1). The traditional leftist value of supporting income redistribution does temper left-wing xenophobia, but less so than expected. On the far right of the political spectrum, in contrast, we find the reverse effect of supporting income redistribution on extreme xenophobia. Other things equal, the chance of being extremely xenophobic of an extremely right-wing person increases from 5.3 to 7.4% when s/he supports income redistribution. The difference is also only marginally significant (*p* < 0.1). Despite the weak effect of supporting redistributive measures on anti-immigrant attitudes, the opposite effects among the two ideological groupings cancel out any net ideological difference in the chance of being xenophobic because of one’s position on income redistribution.

#### The Effect of Egalitarianism

If support for income redistribution only marginally decreases the chance of an extremely left-wing person being extremely xenophobic, do egalitarian values have a significant effect? Other things equal, believing that equal treatment and opportunities are important strongly decreases the predicted probability of being extremely xenophobic among both the extreme left and right. For the average person who identifies as extremely left-wing, xenophobia decreases from 9 to 2.6% and for the average person identifying as extremely right from 14.3 to 5.8% when equality is very important to them. How can egalitarian values have such as strong effect on anti-immigrant attitudes, while support for income redistribution does not? Welfare chauvinism may explain why.

#### The Effect of Welfare Chauvinism

**FIGURE 6:**
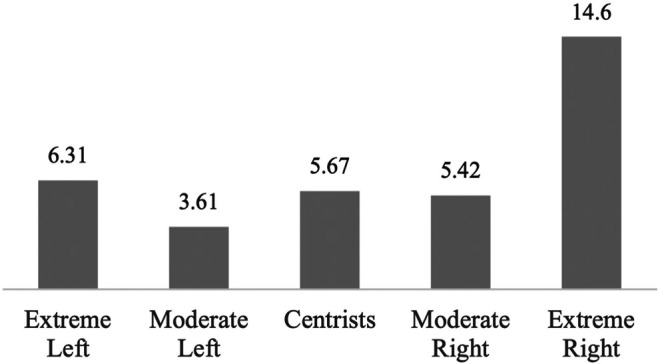
Percentage Welfare Chauvinists Who State that Immigrants Should Never be Granted Social rights by Political Ideology. Source: European Social Survey, cumulative dataset, waves 4 and 8 (N: 41,618). Own calculations.

In the ESS module of waves 4 and 8 (2008 and 2016), there is a direct measure of welfare chauvinism – that immigrants should never be granted social rights – although the pooled sample of these two waves is smaller than the one analyzed so far. Comparing the percentage of welfare chauvinists among each of the five ideological groupings reveals the now-familiar curvilinear pattern along the left-right dimension: welfare chauvinism is higher among the extreme left (6.3%) than the moderate left (3.6%) but similar to the level of the moderate right (5.4%) and centrists (5.7%). As expected, it is highest among the extreme right (14.6%) (see Figure 6).

While only 6.3% of the entire extreme left are welfare chauvinists, 28% of extreme left xenophobes are. Among extreme right xenophobes the share of welfare chauvinists is obviously even larger – 40%. Welfare chauvinism thus seems to be strongly related to anti-immigrant attitudes. It may also explain the relatively high support for redistributive measures among the xenophobic far right; they may primarily want to restrict those measures to themselves. A logistic regression analysis (see Table 3) predicting extreme anti-immigrant attitudes with an interaction term between political ideology and welfare chauvinism confirms that the strong effect on the chance of being extremely xenophobic among the extreme left and the extreme right. Other things equal, the probability increases from 2.3 to 13.4% among the extreme left and from 4.2 to 19.5% among the extreme right.

**TABLE 3:** Results from logistic regression on extremely anti-immigrant attitudes, including a control variable measuring welfare chauvinism.

	Full model		Full model, incl. interaction	
	*Odds Ratio*	*SE*	*Odds Ratio*	*SE*
Political orientation (ref.: Centrists)				
Extreme right	2.596^d^	0.41	2.550^d^	0.47
Moderate right	1.026	0.11	0.986	0.12
Moderate left	0.537^d^	0.06	0.474^d^	0.06
Extreme left	1.465^b^	0.25	1.390^a^	0.26
Welfare chauvinism	5.896^d^	0.58	5.020^d^	0.80
Political Ideology^*^Welfare chauvinism (ref.: Centrists^*^Welfare chauvinism)				
Extreme right # Welfare chauvinism			1.103	0.37
Moderate right # Welfare chauvinism			1.193	0.27
Moderate left # Welfare chauvinism			1.654^a^	0.43
Extreme left # Welfare chauvinism			1.296	0.61
Age	1.003	0.00	1.003	0.00
Male	1.07	0.09	1.07	0.09
1st/2nd generation immigrant	0.606^d^	0.09	0.609^d^	0.09
Post-secondary degree	0.559^d^	0.07	0.561^d^	0.07
Labor force status (ref.: Out of the labor force)				
Employed	0.867	0.09	0.868	0.09
Unemployed	1.129	0.23	1.137	0.23
Finding it difficult on current household income	1.653^d^	0.16	1.654^d^	0.16
Trust in people (ref.: Distrustful)				
Neutral	0.656^d^	0.07	0.658^d^	0.07
Trusting	0.508^d^	0.06	0.509^d^	0.06
Level of social activity (ref.: Less than others)				
About the same	0.782^c^	0.07	0.783^c^	0.07
More than others	0.901	0.11	0.909	0.11
Support income redistribution	1.004	0.10	1.005	0.10
Importance of egalitarianism (ref.: Very)				
Important	0.993	0.10	0.991	0.10
Somewhat important	1.136	0.13	1.134	0.13
Not important	1.195	0.24	1.208	0.24
Importance of following traditions and customs (ref.: Very)				
Important	0.597^d^	0.07	0.598^d^	0.07
Somewhat important	0.442^d^	0.05	0.443^d^	0.05
Not important	0.448^d^	0.06	0.452^d^	0.06
Religiosity, (0 not religious) to 10 (very religious)	0.959^c^	0.02	0.958^c^	0.02
Trust in politicians (ref.: No trust at all)				
Distrustful	0.243^d^	0.02	0.243^d^	0.02
Neutral	0.216^d^	0.03	0.216^d^	0.03
Trusting	0.147^d^	0.02	0.147^d^	0.02
Political participation (voted in last national election)	0.817^b^	0.08	0.819^b^	0.08
*Pseudo R* ^*2*^	0.25		0.25	
*Observations*	41,618		41,618	

*Source*: European Social Survey, cumulative dataset, waves 4 and 8.

Notes: The models also control for year and country. SE = Standard Error.

*P*-values:

a *p* < .1,

b *p* < .05,

c *p* < .01,

d *p* < .001

To summarize, egalitarian values, both in general and specifically with respect to granting social benefits, dampened quite a large amount of extreme left-wing xenophobia. But these values also had a large effect on right-wing xenophobia. General support for income redistribution, by contrast, did not have much explanatory power for xenophobia at either political pole.

### The Role of Cultural Factors in Left and Right-Wing Xenophobia

While values such as egalitarianism, solidarity, and internationalism are traditionally associated with the political left, the importance of traditions and customs are more commonly associated with the political right. Yet unexpectedly we found that traditionalism is rather prevalent among the xenophobic far left. Accordingly, it has a significant effect on both left and right-wing xenophobia, even though the effect is stronger among the far right. The predicted probability of being extremely xenophobic is significantly higher when either a far left or far right person thinks that following traditions and customs is very important. Compared to those who find it not important, the predicted probability of xenophobia increases from 2.9 to 5% among the far left and from 5.8 to 10.1% among the far right.

### Unexplained Variance

Overall, the predicted probability of being extremely xenophobic is 8.2% for the extreme left and 13.1% for the extreme right, when only controlling for sociodemographic factors, country, and year. Controlling for all covariates reduces the probability of xenophobia among the far left to 3.5%, or by 57%, and to 6.7% among the far right, or by 49%. There remains some residual variation in anti-immigrant attitudes yet to be explained.

## Discussion

The analysis has confirmed that there are in fact many far-left xenophobes as well as far-right ones. The same bipolar pattern of anti-immigrant attitudes was found in both the European Social Survey and the European Values Study, so we are relatively confident in this finding. All things equal, there are some striking similarities between these extremists, but also important differences at the two poles of the left-right dimension. On the one hand, far-left xenophobes have significantly lower objective and subjective socioeconomic status than their right-wing counterparts, and far left xenophobia is more influenced by education, labor force status, and perceived socioeconomic position than is far right xenophobia. Even as radical right-wing parties have moved to the center on economic issues, far left identity is retained by economically precarious, if xenophobic native Europeans. Far right nationalist anti-immigrant appeals to communists and others on the far left may run up against limits.

On the other hand, contrary to theoretical expectation, extremists at both ends of the political spectrum do not differ very much from each other in terms of material or cultural values, at least compared to their differences with the broad political center. We expected far left xenophobes may not endorse tradition or nationalism, since the political left typically supports egalitarianism, universalism, and solidarity and since populist and extreme right-wing parties focus on conservative cultural messages, such as populist nationalism and ethnocentrism. However, compared to Europeans overall, left-wing xenophobes were more likely to emphasize the importance of following traditions and customs, just less so than far right xenophobes do. Moreover, believing traditions and customs are very important has an independent significant effect on anti-immigrant attitudes for both the far left as well as the far right. Future research should investigate why those on the far left should feel culturally threatened by immigrants, something that is usually attributed to far right populist nationalists.

As expected, the xenophobic extreme left is more in favor of class-related values traditionally associated with the left than the xenophobic extreme right. They are more supportive of income redistribution, egalitarianism, and granting immigrants social benefits than are extreme right xenophobes. However, these values affect both left and right-wing xenophobia, despite the greater socioeconomic deprivation and insecurity of far left xenophobes. Overall, these values only partially temper far left-wing xenophobia. This suggests that extremism, at least directed at immigrants, is characteristic at both poles of the ideological spectrum, perhaps because political extremists in general share populist and authoritarian tendencies.

As welfare chauvinism, or denial of social rights for immigrants, is relatively prevalent among far left xenophobes, left-wing parties – which have already lost a substantial share of their working-class constituency to the extreme and populist right – may try to retain some far left voters by embracing welfare policies that are more restrictive on the basis of national origin. Political messages and policies promoting welfare chauvinism may be more compatible with left-wing party ideology than rhetoric professing commitment to a threatened national and cultural identity. Much as radical right parties moved to the center on economic and welfare issues, left-wing parties may jettison some egalitarian left-wing values in order to compete with them. In the process, this could ultimately increase racial and ethnic inequality in access to welfare state programs.

Even if controlling for these many differences reduces the probability of xenophobia among the far left by about 60 percent, some residual variation in anti-immigrant attitudes is unexplained. This points to the need for future research on what other factors are decisive for those negative attitudes towards immigrants among the far left. One potential explanation of individual attitudes we did not explore here may be contextual. It is possible that more structural conditions in the country account for some additional variation in individual xenophobia. Our analysis also did not control for respondents’ personal contact with immigrants, which may either increase tolerance or exacerbate ethnic conflict. A final possibility is to explore further the finding that left-wing xenophobes are or feel socially very marginalized and have significantly lower levels of social and political trust. They may thus be enticed to support populist far right or far left parties. They are, however, also less likely to vote, with implications for the future of democracy. As political polarization and rising extremism of all kinds increasingly menace contemporary democracies, it is imperative to devote more scholarly attention to attitudes at both poles of the political spectrum.

## Data Availability

Publicly available datasets were analyzed in this study. The ESS cumulative dataset can be found in the repository of the European Social Survey (https://www.europeansocialsurvey.org/downloadwizard/). The EVS 2017 dataset is accessible through the GESIS Data Collection at the GESIS-Leibniz Institute for the Social Sciences (https://europeanvaluesstudy.eu/methodology-data-documentation/survey-2017/full-release-evs2017/).

## Footnotes

1 We use the term “xenophobia” as shorthand for anti-immigrant sentiments, beliefs, or attitudes.

2 The sample in each country is selected by strict random probability methods. They are representative of the entire population in each country, independent of nationality, citizenship, or language. The data is collected in face-to-face interviews.

3 The total number of cases was 255,824 observations, 69,284 were lost after listwise deletion.

4 Design weights correct for the different likelihoods of each respondent to be part of the sample, whereas population size weights are used to correct for the fact that the included countries have different population sizes but similar sample sizes.

5 We replicated our results with a multinomial logistic regression of attitudes towards immigrants measured in five ordinal categories: 1) extremely anti-immigrant, 2) anti-immigrant, 3) neutral, 4) pro-immigrant, 5) extremely pro-immigrant. The results were substantially the same (see Supplementary Table S2).

6
Table 1 lists the characteristics of the average person of the overall sample.

7 Those who replied 0, or “Immigrants make the country a worse place to live,” on the original 11-point scale are considered “extremely xenophobic.” We ran sensitivity analyses with different cut-off points for extreme xenophobia on the scale (e.g., 0 and 1) and the results were substantially the same. We decided to only include those on the very extreme, as the results were stronger. We realize that concern about immigration is not necessarily racist or xenophobic (Dennison and Geddes 2019). As mentioned, we use the term “xenophobic” as shorthand for an extreme belief that immigration is bad for the country.

8 Those who replied 0 on the original 11-point scale are considered “extremely left-wing,” those who replied 10 “extremely right-wing.” We ran sensitivity analyses with different cut-off points along the scale (e.g., 0 and 1 for far left and 9 and 10 for the far right) and the results were substantially the same. We decided to only include those on the very extreme, as the results were stronger.

9 These were the best available in all countries in all waves. Income in deciles, which makes it comparable across countries, is only available starting in wave 4 (2008), and occupation is not available for those never in the labor force and refers only to last occupation for those currently out of the labor force.

10 A description of all variables included in the models are available in Supplementary Table S1.

11 A similar pattern is revealed in a multinomial logistic regression predicting attitudes towards immigrants (see Supplementary Table S2).

12 See Supplementary Figures 1–3.

13 The results of all interaction effects and the predicted probabilities are documented in Supplementary Tables S3–S13.
