# Supplementary material for: Left-Wing Xenophobia in Europe
Source: Front Sociol. 2021 Jun 10;6:666717. doi: 10.3389/fsoc.2021.666717 (PMC8222516; doi:10.3389/fsoc.2021.666717)

Table 1: List of Variables Used in the Regression Predicting Extremely Anti-Immigrant Attitudes (European Social Survey, cumulative dataset, waves 1-9)

Variables	Description	Question(s) based on	Values
Extreme Anti-Immigrant Attitudes	Whether the respondent believes that immigrants make their country of residence a worse place to live. The original 11-point Likert scale was recoded as follows: 0= extremely xenophobic, 1-10: not extremely xenophobic	Is [country] made a worse or a better place to live by people coming to live here from other countries? (imwbcnt)	Dichotomous (recoded)<br>Values:<br>0= not extremely xenophobic<br>1= extremely xenophobic
Political Ideological Orientation	Self-placement on the political left right scale. The original 11-point Likert scale was recoded as follows: 0= extremely right, 1-4= moderately right, 5= centrist, 6-9= moderately left, 10= extremely left	In politics people sometimes talk of "left" and "right". Using this card, where would you place yourself on this scale, where 0 means the left and 10 means the right? (lrscale)	Categorical (recoded)<br>Values: centrist (reference cat.), extremely right, moderately right, moderately left, extremely left
Age	The respondent's age in years	Age of respondent, calculated (agea)	Continuous (14-114)
Male	Gender of the respondent, coded by the interviewer	Code sex, respondent (gndr)	Dichotomous (recoded)<br>0= female, 1= male
Immigrant Background	Whether the respondent is a first or second generation immigrant (whether either the respondent or his/her mother or father were not born in the country of residence)	Were you born in [country]? (brncntr); Was your father born in [country]? (facntr); Was your mother born in [country]? (mocntr)	Dichotomous (recoded)<br>0= native born; 1= immigrant
Education	Whether the respondent has a post-secondary degree (short-cycle tertiary education and above, ISCED level 5)	What is the highest level of education you have achieved? (edulvla and edulvlb)	Dichotomous (recoded)<br>0= no post-secondary degree<br>1= having a post-secondary
Labor Force Status	Describes the respondent's main activity. "Out of the labor force" includes respondents who are in education, unemployed and not looking for job, permanently sick or disabled, retired, or doing community or military service, or housework	Which of these descriptions best describes your situation (in the last seven days)? (mnactic)	Categorical (recoded)<br>Values: 0=out of the labor force, 1= employed, 2=unemployed
Subjective Socioeconomic Well-Being	Feeling about current household's income. Those who are coded 1 find it either difficult or very difficult to live on the current household income	Which of the descriptions on this card comes closest to how you feel about your household's income nowadays? (hincfel)	Dichotomous (recoded)<br>0= not finding it difficult<br>1= finding it difficult
Support of Income Redistribution	Agreement with the statement that the government should reduce differences in income levels. Those who are coded 1 either agree or strongly agree with the statement.	Please say to what extent you agree or disagree with each of the following statements "The government should take measures to reduce differences in income levels". (gincdif)	Dichotomous (recoded)<br>0= reject income redistribution<br>1=support income redistribution
Importance of Egalitarianism	The respondent's attitudes towards equal treatment and opportunity. The category of respondents who find egalitarianism "somewhat important" includes those who replied "somewhat" and "a little like me," and the category "not important" those who replied "not like me" and "not like me at all."	Now I will briefly describe some people. Please (...) tell me how much each person is or is not like you. "She/he thinks it is important that every person in the world should be treated equally. She/he believes everyone should have equal opportunities in life." (ipeqopt)	Categorical (recoded)<br>Values: 0=very important, 1=important, 2=somewhat important, 3=not important

Welfare Chauvinism	The respondent's attitudes towards granting social benefits and services to immigrants. Those who are coded 1 think that immigrants should never get the same rights as citizens.	Thinking of people coming to live in [country] from other countries, when do you think they should obtain the same rights to social benefits and services as citizens already living here? Please choose the option on this card that comes closest to your view. (imsclbn)	Dichotomous (recoded)<br>0= not a welfare chauvinist<br>1= welfare chauvinist
Importance of Following Traditions and Customs	The importance the respondent assigns to following traditions and customs. The category "somewhat important" includes those who replied "somewhat" and "a little like me," and the category "not important" those who replied "not like me" and "not like me at all."	Now I will briefly describe some people. Please listen to each description and tell me how much each person is or is not like you. "Tradition is important to her/him. She/he tries to follow the customs handed down by her/his religion or her/his family." (imptrad)	Categorical (recoded)<br>Values: 0=very important, 1=important, 2=somewhat important, 3=not important
Religiosity	Self-reported religiosity, independent of whether the respondent identifies as belonging to a religion or denomination or not.	C13. Regardless of whether you belong to a particular religion, how religious would you say you are? (rlgdgr)	Continuous: 0-10
Social Trust	How much trust the repsondent has in other people.	Generally speaking, would you say that most people can be trusted, or that you can't be too careful in dealing with people? Please tell me on a score of 0 to 10, where 0 means you can't be too careful and 10 means that most people can be trusted. (ppltrst)	Categorical (recoded)<br>Values: 0=distrustful, 1=neutral, 2=trusting
Social Activity	Self-reported participation in social activities compared to others of same age	Compared to other people of your age, how often would you say you take part in social activities ? (sclact)	Categorical (recoded)<br>Values: 0=less than others, 1= about the same, 2=more than others
Trust in Politicians	Whether the respondent trusts politicians. The 11-points Likert scale was recoded into a categorical variable as follows: 0= no trust at all, 1-4= distrustful , 5= neutral, 6-10= trust in politicians.	Please tell me on a score of 0-10 how much you personally trust each of the institutions I read out. 0 means you do not trust an institution at all, and 10 means you have complete trust. Firstly...... politicians?	Categorical (recoded)<br>Values: 0=no trust at all, 1=distrustful, 2=neutral, 3=trust in politicians
Political Participation	Whether the respondent voted in the last national elections in his/her country of residence.	Some people don't vote nowadays for one reason or another. Did you vote in the last [country] national election in [month/year]? (vote)	Dichotomous (recoded)<br>0= did not vote; 1= voted
Year	Year of the survey	ESS round (essround)	Categorical: 1-9 (2002-2018)
Country	Respondent's country of residence (country where the data was collected	Country (cntry)	Categorical<br>Values: 0=France, 1=Switzerland, 2=Germany, 3=Spain, 4=Finland, 5=Belgium, 6=Great Britain, 7=Hungary, 8=Ireland, 9=the Netherlands, 10=Norway, 11=Poland, 12=Portugal, 13=Sweden, 14=Slovenia

Table 2: Results from Multinomial Logistic Regression on Attitudes Towards Immigrants, Results Predicting Extreme Xenophobia (base category: being neutral)*

	Full Model	
	Odds Ratio	*SE*
Extremely Anti-Immigrant		
Political Orientation (ref.: Centrists)		
Extreme Right	4.407***	0.359
Moderate Right	1.223***	0.061
Moderate Left	0.652***	0.035
Extreme Left	1.727***	0.142
Age	1.010***	0.001
1st/2nd Generation Immigrant	0.679***	0.046
Male	1.035	0.041
Post-Secondary Degree	0.442***	0.026
Labor Force Status (ref.: Out of the Labor Force)		
Employed	0.892*	0.042
Unemployed	1.298**	0.122
Finding it Difficult on Current Household Income	1.790***	0.081
Trust in People (ref.: Distrustful)		
Neutral	0.569***	0.028
Trusting	0.477***	0.024
Level of Social Activity (ref.: Less than Others)		
About the Same	0.824***	0.035
More than Others	0.851**	0.049
Support Income Redistribution	1.158**	0.055
Importance of Egalitarianism (ref.: very)		
Important	0.987	0.046
Somewhat Important	1.296***	0.071
Not Important	3.015***	0.275
Importance of Following Traditions and Customs (ref.: very)		
Important	0.611***	0.032
Somewhat Important	0.430***	0.024
Not Important	0.547***	0.037
Subjective Religiosity (0-not religious to 10-very religious)	0.959***	0.007
Trust in Politicians (ref.: No Trust at all)		
Distrustful	0.244***	0.011
Neutral	0.165***	0.011
Trusting	0.122***	0.01
Political Participation (voted in last national election)	0.791***	0.037
Anti-Immigrant		
Political Orientation (ref.: Centrists)		
Extreme Right	1.720***	0.096
Moderate Right	1.422***	0.032
Moderate Left	0.901***	0.021
Extreme Left	0.741***	0.042
Age	1.008***	0.001
1st/2nd Generation Immigrant	0.828***	0.025
Male	0.961*	0.017
Post-Secondary Degree	0.623***	0.014
Labor Force Status (ref.: Out of the Labor Force)		
Employed	0.982	0.021
Unemployed	0.996	0.05

Finding it Difficult on Current Household Income	1.256***	0.03
Trust in People (ref.: Distrustful)		
Neutral	0.704***	0.016
Trusting	0.622***	0.014
Level of Social Activity (ref.: Less than Others)		
About the Same	0.910***	0.018
More than Others	0.944*	0.025
Support Income Redistribution	1.126***	0.023
Importance of Egalitarianism (ref.: very)		
Important	1.178***	0.025
Somewhat Important	1.425***	0.036
Not Important	1.859***	0.102
Importance of Following Traditions and Customs (ref.: very)		
Important	0.872***	0.023
Somewhat Important	0.740***	0.02
Not Important	0.698***	0.024
Subjective Religiosity (0-not religious to 10-very religious)	0.985***	0.003
Trust in Politicians (ref.: No Trust at all)		
Distrustful	0.936*	0.025
Neutral	0.614***	0.02
Trusting	0.626***	0.022
Political Participation (voted in last national election)	0.910***	0.021
Neutral *(base category)*		
Pro-Immigrant		
Political Orientation (ref.: Centrists)		
Extreme Right	1.02	0.06
Moderate Right	1.178***	0.026
Moderate Left	1.510***	0.032
Extreme Left	1.494***	0.076
Age	0.996***	0.001
1st/2nd Generation Immigrant	1.413***	0.037
Male	1.080***	0.019
Post-Secondary Degree	1.330***	0.025
Labor Force Status (ref.: Out of the Labor Force)		
Employed	1.041*	0.021
Unemployed	1.036	0.052
Finding it Difficult on Current Household Income	0.932**	0.023
Trust in People (ref.: Distrustful)		
Neutral	1	0.024
Trusting	1.559***	0.033
Level of Social Activity (ref.: Less than Others)		
About the Same	1.042*	0.02
More than Others	1.200***	0.03
Support Income Redistribution	1.124***	0.022
Importance of Egalitarianism (ref.: very)		
Important	0.817***	0.016
Somewhat Important	0.651***	0.016
Not Important	0.753***	0.044
Importance of Following Traditions and Customs (ref.: very)		
Important	1.052+	0.028
Somewhat Important	1.142***	0.031

Not Important	1.281***	0.043
Subjective Religiosity (0-not religious to 10-very religious)	1.022***	0.003
Trust in Politicians (ref.: No Trust at all)		
Distrustful	1.285***	0.038
Neutral	1.448***	0.049
Trusting	2.062***	0.07
Political Participation (voted in last national election)	1.100***	0.026
Extremely Pro-Immigrant		
Political Orientation (ref.: Centrists)		
Extreme Right	2.585***	0.283
Moderate Right	0.788***	0.053
Moderate Left	1.656***	0.091
Extreme Left	4.464***	0.369
Age	0.989***	0.001
1st/2nd Generation Immigrant	2.193***	0.12
Male	1.173***	0.052
Post-Secondary Degree	1.745***	0.08
Labor Force Status (ref.: Out of the Labor Force)		
Employed	0.962	0.049
Unemployed	1.199	0.135
Finding it Difficult on Current Household Income	1.112+	0.067
Trust in People (ref.: Distrustful)		
Neutral	0.985	0.064
Trusting	1.602***	0.088
Level of Social Activity (ref.: Less than Others)		
About the Same	0.989	0.05
More than Others	1.555***	0.092
Support Income Redistribution	1.221***	0.064
Importance of Egalitarianism (ref.: very)		
Important	0.413***	0.02
Somewhat Important	0.294***	0.022
Not Important	0.509***	0.077
Importance of Following Traditions and Customs (ref.: very)		
Important	0.718***	0.048
Somewhat Important	0.826**	0.057
Not Important	1.535***	0.115
Subjective Religiosity (0-not religious to 10-very religious)	1.015	0.009
Trust in Politicians (ref.: No Trust at all)		
Distrustful	0.675***	0.044
Neutral	0.761***	0.061
Trusting	1.223**	0.095
Political Participation (voted in last national election)	1.247***	0.075
Pseudo R^2	0.104	
Observations	186,540	

Source: European Social Survey, cumulative dataset, waves 1-9.

Notes: The models also control for year and country. SE = Standard Error. p-values: + p < .1, * p < .05, ** p < .01, *** p < .001

Table 3: Interaction Effect of Political Orientation and Education from Logistic Regression on Extremely Anti-Immigrant Attitudes

	Odds Ratio	*SE*
Political Orientation (ref.: Centrists)		
Extreme Right	3.20***	0.249
Moderate Right	1.01	0.052
Moderate Left	0.63***	0.035
Extreme Left	1.68***	0.134
Post-Secondary Degree	0.49***	0.047
Interaction of Political Ideology*Post-Secondary Degree		
Extreme Right*Post-Secondary Degree	1.42	0.309
Moderate Right*Post-Secondary Degree	0.99	0.137
Moderate Left*Post-Secondary Degree	0.76+	0.119
Extreme Left*Post-Secondary Degree	0.82	0.182
Pseudo R^2	0.192	
Observations	186,540	

Source: European Social Survey, cumulative dataset, waves 1-9.

Notes: The model controls for all variables included in the logistic regression on extremely anti-immigrant attitudes reported in Table 2. SE = Standard Error. p-values: $^{+}$ p < .1, * p < .05, ** p < .01, *** p

Table 3.1.: Predicted Probabilities of Being Extremely Xenophobic by Political Orientation and Education

	Margin	*SE*	*z*	*P>z*	*95% Confidence Interval*		*90% Confidence Interval*	
Extreme Right*No Post-Secondary Degree	0.081	0.005	15.05	0.000	0.070	0.091	0.072	0.089
Extreme Right*Post-Secondary Degree	0.058	0.010	5.70	0.000	0.038	0.078	0.041	0.074
Extreme Left*No Post-Secondary Degree	0.044	0.003	13.67	0.000	0.038	0.050	0.039	0.049
Extreme Left*Post-Secondary Degree	0.018	0.003	5.37	0.000	0.012	0.025	0.013	0.024

Note: The model calculates the predicted probabilities for the average person with the characteristics in Table 1, controlling for all variables included in the logistic regression on extremely anti-immigrant attitudes reported in Table 2.

Table 4: Interaction Effect of Political Orientation and Labor Force Status from Logistic Regression on Extremely Anti-Immigrant Attitudes

	Odds Ratio	*SE*
Political Orientation (ref.: Centrists)		
Extreme Right	3.11***	0.299
Moderate Right	0.93	0.063
Moderate Left	0.73***	0.052
Extreme Left	2.01***	0.201
Labor Force Status (ref.: Out of the Labor Force)		
Employed	0.95	0.062
Unemployed	1.33*	0.179
Political Orientation*Labor Force Status		
Extreme Right*Employed	1.21	0.180
Extreme Right*Unemployed	0.87	0.285
Moderate Right*Employed	1.13	0.108
Moderate Right*Unemployed	1.53+	0.347
Moderate Left*Employed	0.67***	0.071
Moderate Left*Unemployed	0.8	0.167
Extreme Left*Employed	0.71*	0.109
Extreme Left*Unemployed	0.47**	0.134
Pseudo R^2	0.193	
Observations	186,540	

Source: European Social Survey, cumulative dataset, waves 1-9.
Notes: The model controls for all variables included in the logistic regression on extremely anti-immigrant attitudes reported in Table 2. SE = Standard Error. p-values: $^{+}$ p < .1, * p < .05, ** p < .01, *** p

Table 4.1.: Predicted Probabilities of Being Extremely Xenophobic by Political Orientation and Labor Force Status

	Margin	*SE*	*z*	*P>z*	*95% Confidence Interval*		*90% Confidence Interval*	
Extreme Right*Out of the Labor Force	0.065	0.005	12.01	0.000	0.054	0.075	0.056	0.074
Extreme Right*Employed	0.074	0.007	9.87	0.000	0.059	0.088	0.061	0.086
Extreme Right*Unemployed	0.074	0.020	3.71	0.000	0.035	0.113	0.041	0.107
Extreme Left*Out of the Labor Force	0.043	0.004	11.14	0.000	0.035	0.050	0.037	0.049
Extreme Left*Employed	0.029	0.003	9.03	0.000	0.023	0.035	0.024	0.034
Extreme Left*Unemployed	0.027	0.006	4.25	0.000	0.015	0.040	0.017	0.037

Note: The model calculates the predicted probabilities for the average person with the characteristics in Table 1, controlling for all variables included in the logistic regression on extremely anti-immigrant attitudes reported in Table 2.

Table 5: Interaction Effect of Political Orientation and Perceived Socioeconomic Position from Logistic Regression on Extremely Anti-Immigrant Attitudes

	Odds Ratio	*SE*
Political Orientation (ref.: Centrists)		
Extreme Right	3.407***	0.292
Moderate Right	0.969	0.055
Moderate Left	0.590***	0.038
Extreme Left	1.666***	0.158
Finding it Difficult on Current Household Income (ref.: not finding it difficult)	1.578***	0.102
Interaction of Political Ideology*Perceived Socioeconomic Position		
Extreme Right*Difficult on HH income	0.915	0.145
Moderate Right*Difficult on HH income	1.155	0.117
Moderate Left*Difficult on HH income	1.078	0.116
Extreme Left*Difficult on HH income	0.977	0.147
Pseudo R^2	0.191	
Observations	186,540	

Source: European Social Survey, cumulative dataset, waves 1-9.

Notes: The model controls for all variables included in the logistic regression on extremely anti-immigrant attitudes reported in Table 2. SE = Standard Error. p-values: $^{+}$ p < .1, * p < .05, ** p < .01, *** p

Table 5.1.: Predicted Probabilities of Being Extremely Xenophobic by Political Orientation and Perceived Socioeconomic Position

	Margin	*SE*	*z*	*P>z*	*95% Confidence Interval*		*90% Confidence Interval*	
Extreme Right*Not Difficult on HH Income	0.065	0.005	13.51	0.000	0.055	0.074	0.057	0.072
Extreme Right*Difficult on HH Income	0.091	0.011	8.55	0.000	0.070	0.111	0.073	0.108
Extreme Left*Not Difficult on HH Income	0.033	0.003	11.57	0.000	0.027	0.038	0.028	0.037
Extreme Left*Difficult on HH Income	0.049	0.005	9.47	0.000	0.039	0.060	0.041	0.058

Note: The model calculates the predicted probabilities for the average person with the characteristics in Table 1, controlling for all variables included in the logistic regression on extremely anti-immigrant attitudes reported in Table 2.

Table 6: Interaction Effect of Political Orientation and Social Activity from Logistic Regression on Extremely Anti-Immigrant Attitudes

	Odds Ratio	*SE*
Political Orientation (ref.: Centrists)		
Extreme Right	2.958***	0.312
Moderate Right	0.979	0.068
Moderate Left	0.647***	0.047
Extreme Left	1.674***	0.177
Level of Social Activity (ref.: Less than Others)		
About the Same	0.811**	0.053
More than Others	0.938	0.085
Political Orientation*Social Activity		
Extreme Right*About the Same	1.272	0.197
Extreme Right*More than Others	1.098	0.224
Moderate Right*About the Same	1.132	0.115
Moderate Right*More than Others	0.867	0.117
Moderate Left*About the Same	0.95	0.105
Moderate Left*More than Others	0.715*	0.112
Extreme Left*About the Same	1.129	0.181
Extreme Left*More than Others	0.658+	0.143
Pseudo R^2	0.192	
Observations	186,540	

Source: European Social Survey, cumulative dataset, waves 1-9.

Notes: The model controls for all variables included in the logistic regression on extremely anti-immigrant attitudes reported in Table 2. SE = Standard Error. p-values: $^{+}$ p < .1, * p < .05, ** p < .01, *** p

Table 6.1.: Predicted Probabilities of Being Extremely Xenophobic by Political Orientation and Social Activity

	Margin	*SE*	*z*	*P>z*	*95% Confidence Interval*		*90% Confidence Interval*	
Extreme Right*Less Social Activity	0.067	0.006	10.90	0.000	0.055	0.079	0.057	0.077
Extreme Right*About the Same	0.069	0.007	9.94	0.000	0.055	0.083	0.058	0.080
Extreme Right*More Social Activity	0.069	0.010	6.78	0.000	0.049	0.089	0.052	0.086
Extreme Left*Less Social Activity	0.039	0.004	10.45	0.000	0.032	0.046	0.033	0.045
Extreme Left*About the Same	0.036	0.004	9.01	0.000	0.028	0.044	0.029	0.042
Extreme Left*More Social Activity	0.024	0.004	5.84	0.000	0.016	0.033	0.018	0.031

Note: The model calculates the predicted probabilities for the average person with the characteristics in Table 1, controlling for all variables included in the logistic regression on extremely anti-immigrant attitudes reported in Table 2.

Table 7: Interaction Effect of Political Orientation and Social Trust from Logistic Regression on Extremely Anti-Immigrant Attitudes

	Odds Ratio	*SE*
Political Orientation (ref.: Centrists)		
Extreme Right	3.018***	0.290
Moderate Right	0.985	0.064
Moderate Left	0.649***	0.044
Extreme Left	1.942***	0.188
Trust in People (ref.: Distrustful)		
Neutral	0.658***	0.048
Trusting	0.554***	0.043
Political Orientation*Trust in People		
Extreme Right*Neutral	1.276	0.225
Extreme Right*Trusting	1.23	0.218
Moderate Right*Neutral	1.131	0.133
Moderate Right*Trusting	0.974	0.110
Moderate Left*Neutral	0.955	0.126
Moderate Left*Trusting	0.742*	0.096
Extreme Left*Neutral	0.703+	0.135
Extreme Left*Trusting	0.576**	0.111
Pseudo R^2	0.192	
Observations	186,540	

Source: European Social Survey, cumulative dataset, waves 1-9.

Notes: The model controls for all variables included in the logistic regression on extremely anti-immigrant attitudes reported in Table 2. SE = Standard Error. p-values: $^{+}p < .1$, $^{*}p < .05$, $^{**}p < .01$, *** p

Table 7.1.: Predicted Probabilities of Being Extremely Xenophobic by Political Orientation and Social Trust

	Margin	*SE*	*z*	*P>z*	*95% Confidence Interval*		*90% Confidence Interval*	
Extreme Right*Distrustful	0.086	0.007	12.26	0.000	0.073	0.100	0.075	0.098
Extreme Right*Neutral	0.074	0.009	7.86	0.000	0.055	0.092	0.058	0.089
Extreme Right*Trusting	0.061	0.008	7.73	0.000	0.045	0.076	0.048	0.073
Extreme Left*Distrustful	0.057	0.005	11.52	0.000	0.048	0.067	0.049	0.066
Extreme Left*Neutral	0.027	0.004	6.49	0.000	0.019	0.036	0.020	0.034
Extreme Left*Trusting	0.019	0.003	6.48	0.000	0.013	0.025	0.014	0.024

Note: The model calculates the predicted probabilities for the average person with the characteristics in Table 1, controlling for all variables included in the logistic regression on extremely anti-immigrant attitudes reported in Table 2.

Table 8: Interaction Effect of Political Orientation and Political Trust from Logistic Regression on Extremely Anti-Immigrant Attitudes

	Odds Ratio	*SE*
Political Orientation (ref.: Centrists)		
Extreme Right	2.965***	0.313
Moderate Right	0.958	0.073
Moderate Left	0.594***	0.046
Extreme Left	1.353**	0.133
Trust in Politicians (ref.: No Trust at all)		
Distrustful	0.223***	0.015
Neutral	0.176***	0.018
Trusting	0.104***	0.015
Interaction of Political Ideology and Trust in Politicians		
Extreme Right*Distrustful	1.285	0.207
Extreme Right*Neutral	1.038	0.248
Extreme Right*Trusting	1.382	0.343
Moderate Right*Distrustful	1.119	0.118
Moderate Right*Neutral	1.013	0.158
Moderate Right*Trusting	1.143	0.217
Moderate Left*Distrustful	1.039	0.116
Moderate Left*Neutral	1.099	0.195
Moderate Left*Trusting	0.964	0.221
Extreme Left*Distrustful	1.403*	0.224
Extreme Left*Neutral	1.677*	0.433
Extreme Left*Trusting	2.708***	0.756
Pseudo R^2	0.192	
Observations	186,540	

Source: European Social Survey, cumulative dataset, waves 1-9.

Notes: The model controls for all variables included in the logistic regression on extremely anti-immigrant attitudes reported in Table 2. SE = Standard Error. p-values: $^{+}$ p < .1, * p < .05, ** p < .01, *** p

Table 8.1.: Predicted Probabilities of Being Extremely Xenophobic by Political Orientation and Political Trust

	Margin	*SE*	*z*	*P>z*	*95% Confidence Interval*		*90% Confidence Interval*	
Extreme Right*No Trust at all	0.215	0.016	13.08	0.000	0.183	0.247	0.188	0.242
Extreme Right*Distrustful	0.073	0.008	9.41	0.000	0.057	0.088	0.060	0.085
Extreme Right*Neutral	0.048	0.009	5.32	0.000	0.030	0.065	0.033	0.062
Extreme Right*Trusting	0.038	0.007	5.76	0.000	0.025	0.051	0.027	0.049
Extreme Left*Not Trust at all	0.111	0.009	12.32	0.000	0.093	0.129	0.096	0.126
Extreme Left*Distrustful	0.038	0.004	8.76	0.000	0.029	0.046	0.030	0.045
Extreme Left*Neutral	0.036	0.008	4.65	0.000	0.021	0.051	0.023	0.048
Extreme Left*Trusting	0.034	0.007	4.61	0.000	0.020	0.049	0.022	0.046

Note: The model calculates the predicted probabilities for the average person with the characteristics in Table 1, controlling for all variables included in the logistic regression on extremely anti-immigrant attitudes reported in Table 2.

Table 9: Interaction Effect of Political Orientation and Political Participation from Logistic Regression on Extremely Anti-Immigrant Attitudes

	Odds Ratio	*SE*
Political Orientation (ref.: Centrists)		
Extreme Right	3.630***	0.610
Moderate Right	1.137	0.109
Moderate Left	0.641***	0.064
Extreme Left	1.629***	0.221
Voted in Last National Election (ref.: did not vote)	0.851*	0.055
Interaction of Political Ideology and Voted in Last National Elections		
Extreme Right*Voted	0.887	0.164
Moderate Right*Voted	0.85	0.093
Moderate Left*Voted	0.92	0.107
Extreme Left*Voted	1.014	0.163
Pseudo R^2	0.191	
Observations	186,540	

Source: European Social Survey, cumulative dataset, waves 1-9.

Notes: The model controls for all variables included in the logistic regression on extremely anti-immigrant attitudes reported in Table 2. SE = Standard Error. p-values: $^{+}$ p < .1, * p < .05, ** p < .01, *** p

Table 9.1.: Predicted Probabilities of Being Extremely Xenophobic by Political Orientation and Political Participation

	Margin	*SE*	*z*	*P>z*	*95% Confidence Interval*		*90% Confidence Interval*	
Extreme Right*Did not Vote	0.083	0.012	6.78	0.000	0.059	0.107	0.063	0.103
Extreme Right*Voted	0.064	0.004	14.25	0.000	0.055	0.073	0.057	0.071
Extreme Left*Did not Vote	0.039	0.005	8.07	0.000	0.030	0.049	0.031	0.047
Extreme Left*Voted	0.034	0.003	12.44	0.000	0.029	0.039	0.029	0.038

Note: The model calculates the predicted probabilities for the average person with the characteristics in Table 1, controlling for all variables included in the logistic regression on extremely anti-immigrant attitudes reported in Table 2.

Table 10: Interaction Effect of Political Orientation and Support of Income Redistribution from Logistic Regression on Extremely Anti-Immigrant Attitudes

	Odds Ratio	*SE*
Political Orientation (ref.: Centrists)		
Extreme Right	2.729***	0.387
Moderate Right	1.044	0.094
Moderate Left	0.635***	0.073
Extreme Left	2.691***	0.483
Support of Income Redistribution	1.095	0.079
Interaction of Political Ideology*Support Income Redistribution		
Extreme Right*Support Income Redistribution	1.308+	0.213
Moderate Right*Support Income Redistribution	0.953	0.100
Moderate Left*Support Income Redistribution	0.94	0.120
Extreme Left*Support Income Redistribution	0.556**	0.109
Pseudo R^2	0.192	
Observations	186,540	

Source: European Social Survey, cumulative dataset, waves 1-9.

Notes: The model controls for all variables included in the logistic regression on extremely anti-immigrant attitudes reported in Table 2. SE = Standard Error. p-values: $^{+}$ p < .1, * p < .05, ** p < .01, *** p

Table 10.1.: Predicted Probabilities of Being Extremely Xenophobic by Political Orientation and Support of Income Redistribution

	Margin	*SE*	*z*	*P>z*	*95% Confidence Interval*		*90% Confidence Interval*	
Extreme Right*No Support of Income Redistribution	0.053	0.006	8.26	0.000	0.040	0.065	0.042	0.063
Extreme Right*Support Income Redistribution	0.074	0.005	13.52	0.000	0.063	0.084	0.065	0.083
Extreme Left*No Support of Income Redistribution	0.052	0.008	6.23	0.000	0.036	0.068	0.038	0.066
Extreme Left*Support Income Redistribution	0.032	0.002	13.19	0.000	0.028	0.037	0.028	0.036

Note: The model calculates the predicted probabilities for the average person with the characteristics in Table 1, controlling for all variables included in the logistic regression on extremely anti-immigrant attitudes reported in Table 2.

Table 11: Interaction Effect of Political Orientation and Importance of Egalitarianism from Logistic Regression on Extremely Anti-Immigrant Attitudes

	Odds Ratio	*SE*
Political Orientation (ref.: Centrists)		
Extreme Right	2.769***	0.341
Moderate Right	1.026	0.087
Moderate Left	0.502***	0.044
Extreme Left	1.226+	0.133
Importance of Egalitarianism (ref.: very)		
Important	0.857*	0.060
Somewhat Important	1.065	0.090
Not Important	2.746***	0.369
Interaction of Political Ideology and Importance of Egalitarianism		
Extreme Right*Important	1.157	0.196
Extreme Right*Somewhat Important	1.730**	0.338
Extreme Right*Not Important	0.997	0.269
Moderate Right*Important	1.095	0.122
Moderate Right*Somewhat Important	0.955	0.122
Moderate Right*Not Important	0.641*	0.130
Moderate Left*Important	1.344*	0.159
Moderate Left*Somewhat Important	1.522**	0.213
Moderate Left*Not Important	0.882	0.223
Extreme Left*Important	1.781***	0.291
Extreme Left*Somewhat Important	1.873**	0.424
Extreme Left*Not Important	1.318	0.508
Pseudo R^2	0.193	
Observations	186,540	

Source: European Social Survey, cumulative dataset, waves 1-9.
Notes: The model controls for all variables included in the logistic regression on extremely anti-immigrant attitudes reported in Table 2. SE = Standard Error. p-values: $^{+}$ p < .1, * p < .05, ** p < .01, *** p

Table 11.1.: Predicted Probabilities of Being Extremely Xenophobic by Political Orientation and Importance of Egalitarianism

	Margin	*SE*	*z*	*P>z*	*95% Confidence Interval*		*90% Confidence Interval*	
Extreme Right*Very Important	0.058	0.006	9.33	0.000	0.045	0.070	0.047	0.068
Extreme Right*Important	0.057	0.006	9.55	0.000	0.045	0.069	0.047	0.067
Extreme Right*Somewhat Important	0.101	0.013	7.98	0.000	0.076	0.126	0.080	0.122
Extreme Right*Not Important	0.143	0.025	5.63	0.000	0.093	0.193	0.101	0.185
Extreme Left*Very Important	0.026	0.003	10.35	0.000	0.021	0.031	0.022	0.031
Extreme Left*Important	0.040	0.004	8.91	0.000	0.031	0.048	0.032	0.047
Extreme Left*Somewhat Important	0.051	0.009	5.56	0.000	0.033	0.069	0.036	0.066
Extreme Left*Not Important	0.089	0.028	3.14	0.002	0.034	0.145	0.042	0.136

Note: The model calculates the predicted probabilities for the average person with the characteristics in Table 1, controlling for all variables included in the logistic regression on extremely anti-immigrant attitudes reported in Table 2.

Table 12: Interaction Effect of Political Orientation and Importance of Traditionalism from Logistic Regression on Extremely Anti-Immigrant Attitudes

	Odds Ratio	*SE*
Political Orientation (ref.: Centrists)		
Extreme Right	3.158***	0.384
Moderate Right	0.973	0.085
Moderate Left	0.656***	0.067
Extreme Left	1.486**	0.209
Importance of Traditionalism (ref.: very)		
Important	0.616***	0.050
Somewhat Important	0.470***	0.040
Not Important	0.630***	0.060
Interaction of Political Ideology and Importance of Traditionalism		
Extreme Right*Important	1.106	0.190
Extreme Right*Somewhat Important	1.23	0.245
Extreme Right*Not Important	0.863	0.210
Moderate Right*Important	0.986	0.120
Moderate Right*Somewhat Important	1.093	0.136
Moderate Right*Not Important	1.156	0.177
Moderate Left*Important	1.13	0.156
Moderate Left*Somewhat Important	0.822	0.118
Moderate Left*Not Important	0.709*	0.111
Extreme Left*Important	1.292	0.255
Extreme Left*Somewhat Important	1.217	0.243
Extreme Left*Not Important	0.932	0.201
Pseudo R^2	0.192	
Observations	186,540	

Source: European Social Survey, cumulative dataset, waves 1-9.

Notes: The model controls for all variables included in the logistic regression on extremely anti-immigrant attitudes reported in Table 2. SE = Standard Error. p-values: $^{+}p < .1$, $^{*}p < .05$, $^{**}p < .01$, *** p

Table 12.1.: Predicted Probabilities of Being Extremely Xenophobic by Political Orientation and Importance of Traditionalism

	Margin	*SE*	*z*	*P>z*	*95% Confidence Interval*		*90% Confidence Interval*	
Extreme Right*Very Important	0.101	0.010	10.34	0.000	0.082	0.120	0.085	0.117
Extreme Right*Important	0.071	0.008	9.46	0.000	0.056	0.086	0.059	0.084
Extreme Right*Somewhat Important	0.061	0.009	7.09	0.000	0.044	0.078	0.047	0.075
Extreme Right*Not Important	0.058	0.011	5.23	0.000	0.036	0.079	0.039	0.076
Extreme Left*Very Important	0.050	0.006	8.09	0.000	0.038	0.062	0.040	0.060
Extreme Left*Important	0.040	0.005	8.08	0.000	0.031	0.050	0.032	0.049
Extreme Left*Somewhat Important	0.029	0.004	7.73	0.000	0.022	0.037	0.023	0.036
Extreme Left*Not Important	0.030	0.004	6.85	0.000	0.022	0.039	0.023	0.037

Note: The model calculates the predicted probabilities for the average person with the characteristics in Table 1, controlling for all variables included in the logistic regression on extremely anti-immigrant attitudes reported in Table 2.

Table 13: Predicted Probabilities of Being Extremely Xenophobic by Political Orientation and Welfare Chauvinism

	Margin	*SE*	*z*	*P>z*	*95% Confidence Interval*		*90% Confidence Interval*	
Extreme Right*Not a Welfare Chauvinist	0.042	0.007	6.08	0.000	0.028	0.055	0.031	0.053
Extreme Right*Welfare Chauvinist	0.195	0.039	4.95	0.000	0.118	0.272	0.130	0.260
Extreme Left*Not a Welfare Chauvinist	0.023	0.004	5.73	0.000	0.015	0.031	0.017	0.030
Extreme Left*Welfare Chauvinist	0.134	0.049	2.75	0.006	0.039	0.230	0.054	0.215

Note: The model calculates the predicted probabilities for the average person of the sample used in the regression analysis reported in Table 3, controlling for all variables included in the logistic regression on extremely anti-immigrant attitudes reported in Table 3.

Fig. 1: Percentage of Respondents Strongly Agreeing with "Immigrants are a Strain on the Welfare System"
(Source: European Value Survey, integrated dataset, wave 7. Own calculations.)

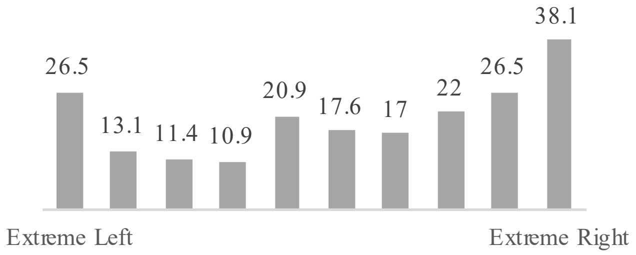

Fig. 2: Percentage of Respondents Strongly Agreeing with "Immigrants Take Away Jobs From (Nationality)"
(Source: European Value Survey, integrated dataset, wave 7. Own calculations.)

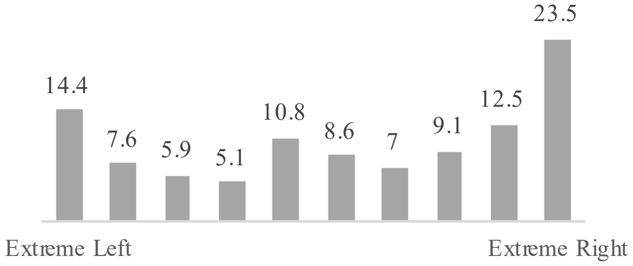

Fig. 3: Percentage of Respondents Strongly Agreeing with "Important that Immigrants do not Maintain Distinct Customs and Traditions"
(Source: European Value Survey, integrated dataset, wave 7. Own calculations.)

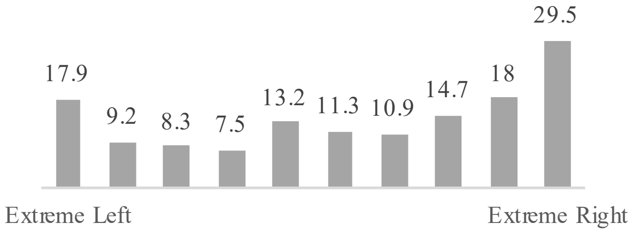

Supplement: Supplementary file 1 [file DataSheet1.pdf]
